# A MALT1 inhibitor suppresses human myeloid DC, effector T-cell and B-cell responses and retains Th1/regulatory T-cell homeostasis

**DOI:** 10.1371/journal.pone.0222548

**Published:** 2020-09-01

**Authors:** Celine Dumont, Ulf Sivars, Theresa Andreasson, Lina Odqvist, Johan Mattsson, Amy DeMicco, Katerina Pardali, Gustav Johansson, Linda Yrlid, Rhona J. Cox, Frank Seeliger, Marie Larsson, Ulf Gehrmann, Andrew M. Davis, Outi Vaarala

**Affiliations:** 1 Research & Early Development, Respiratory, Inflammation & Autoimmune, R&D BioPharmaceuticals, AstraZeneca, Gothenburg, Sweden; 2 Clinical Pharmacology & Safety Sciences, R&D BioPharmaceuticals Gothenburg, Sweden; J. Heyrovsky Institute of Physical Chemistry, CZECH REPUBLIC

## Abstract

The paracaspase mucosa-associated lymphoid tissue lymphoma translocation protein-1 (MALT1) regulates nuclear-factor-kappa-B (NF-κB) activation downstream of surface receptors with immunoreceptor tyrosine-based activation motifs (ITAMs), such as the B-cell or T-cell receptor and has thus emerged as a therapeutic target for autoimmune diseases. However, recent reports demonstrate the development of lethal autoimmune inflammation due to the excessive production of interferon gamma (IFN-ɣ) and defective differentiation of regulatory T-cells in genetically modified mice deficient in MALT1 paracaspase activity. To address this issue, we explored the effects of pharmacological MALT1 inhibition on the balance between T-effector and regulatory T-cells. Here we demonstrate that allosteric inhibition of MALT1 suppressed Th1, Th17 and Th1/Th17 effector responses, and inhibited T-cell dependent B-cell proliferation and antibody production. Allosteric MALT1 inhibition did not interfere with the suppressive function of human T-regulatory cells, although it impaired de novo differentiation of regulatory T-cells from naïve T-cells. Treatment with an allosteric MALT1 inhibitor alleviated the cytokine storm, including IFN-ɣ, in a mouse model of acute T-cell activation, and long-term treatment did not lead to an increase in IFN-ɣ producing CD4 cells or tissue inflammation. Together, our data demonstrate that the effects of allosteric inhibition of MALT1 differ from those seen in mice with proteolytically inactive MALT1, and thus we believe that MALT1 is a viable target for B and T-cell driven autoimmune diseases.

## Introduction

The paracaspase MALT1 is a central signaling protein, which has an essential role in the activation of the transcription factor NF-kappa B (NF-κB) downstream of surface receptors with immunoreceptor tyrosine-based activation motifs (ITAMs), such as the B- or T-cell receptor. MALT1 signalling also regulates dendritic cell (DC) function activated by ITAM-containing receptor Dectin-1 [1,2].

The mechanisms of MALT1-promoted NF-κB activation are based on both its scaffold and enzymatic functions. Upon activation, MALT1 acts as a scaffold in a complex with Carma1-and BCL10 (the CBM complex) and physically recruits the ubiquitin ligase TRAF6, which mediates the recruitment and activation of the I-kappaB kinase (IKK) complex leading to proteasomal degradation of NF-κB inhibitor IκB [3]. The paracaspase activity affects NF-κB through cleavage of several substrates including the adaptor protein BCL10 [4], transcription factor RELB [5], A20 [6,7], CYLD [8], the ubiquitin ligase HOIL1 and also by auto processing of MALT1 [9]. In addition to the effects of MALT1 in promoting NF-κB activity, MALT1 protease cleaves the endoribonucleases Regnase-1 and Roquin [10,11–13], and increases the stability of mRNAs in activated T-cells [14–16]. It has recently been identified that MALT1 cleaves the RNAse N4BP1 in T-cells and macrophages, an interferon-inducible inhibitor of HIV-1 [17].

Based on the central role of MALT1 in lymphocyte activation, MALT1 has been identified as a target for therapeutic immunomodulation in chronic inflammatory or autoimmune diseases. MALT1-deficient mice have been shown to be protected from autoimmune diseases, such as experimental allergic encephalitis [18,19].

The role of the paracaspase activity of MALT1 has been studied *in vivo* using genetically modified knock-in (MALT1-PD) mice expressing an inactive form of the MALT1 protease. Somewhat surprisingly, several independent studies have reported development of spontaneous lethal autoimmune inflammation, such as multi-organ inflammation, gastritis and ataxia, in MALT1-PD mice [20–23]. In these models, the differential functions of MALT1 scaffold and paracaspase activity have been demonstrated, and the net outcome of selective paracaspase inactivation shown to result in the imbalance of the immune homeostasis between effector CD4+ T-cells and regulatory T-cells; excessive production of interferon gamma (IFN-ɣ) and defective differentiation of regulatory T-cells. These reports have raised the concern that pharmacological inhibition of MALT1 could potentially interfere with immune homeostasis and cause a similar kind of severe auto-inflammation as observed in the mice expressing proteolytically inactive MALT1. Due to the complex function of MALT1, as exemplified by the knock-out and MALT1-protease-dead mice, the consequences of pharmacological inhibition of MALT1 are difficult to predict *in vivo*.

To address these issues, we studied the effects of pharmacological MALT1 inhibition in human immune cells *in vitro* using a novel and selective allosteric inhibitor of MALT1. As the effects of small molecule inhibitors are not comparable to complete inhibition of MALT1 protease function seen in genetically modified models, we hypothesized that allosteric inhibition of MALT1 might provide an effective and safe route to down-regulate of B- and T-cell activation without promoting the IFN-γ driven autoinflammatory pathology.

## Materials and methods

Animal experiments were approved by the Local Ethical committee Regionala etikprövningsnämnden Göteborg (123–2014). Mice were euthanized by inhalation of isoflurane and exsanguination. All blood donor volunteers signed an Informed Consent form and donation was approved by AstraZeneca Institutional review board and local Ethics committee Regionala etikprövningsnämnden Göteborg (033–10).

### Chemical synthesis

Mepazine (Compound 1) was synthesized according to the published procedure [24]. Compound 3 was also synthesised according to the published procedure [25]. Compound 2 was synthesised as follows.

Step 1
  Methyl 4-amino-2-chlorobenzoate (1.88 g, 10.2 mmol) was added to a mixture of 1-phenyl-5-(trifluoromethyl)-1*H*-pyrazole-4-carboxylic acid (2.0 g, 7.8 mmol), 1-propanephosphonic acid cyclic anhydride (50% in ethyl acetate) (9.9 g, 15.6 mmol) and *N*,*N*-diisopropylethylamine (4.1 mL, 23.4 mmol) in ethyl acetate (20 mL) under nitrogen. The resulting solution was stirred at 50 °C for 16 h. The reaction mixture was diluted with ethyl acetate and washed sequentially with saturated aqueous sodium hydrogen carbonate (x 2) and saturated brine (x 2). The organic layer was dried over sodium sulphate, filtered and concentrated. The crude product was purified by flash silica chromatography, elution gradient 5 to 50% ethyl acetate in petroleum ether, to afford methyl 2-chloro-4-(1-phenyl-5-(trifluoromethyl)-1*H*-pyrazole-4-carboxamido)benzoate (3.10 g, 94%) as a pale yellow oil which solidified on standing. m/z (ES+), [M+H]+ = 424.1; C18, 10–95% acetonitrile (5mM aqueous NH_4_HCO_3_), 2.6min, HPLC tR = 1.5 min. ^1^H NMR (400 MHz, chloroform-*d*) δ ppm 3.95 (s, 3 H), 7.44–7.51 (m, 2 H) 7.51–7.58 (m, 3 H), 7.60 (dd, 1 H) 7.85 (d, 2 H) 7.93 (d, 1 H) 8.05 (s, 1 H).
Step 2
  2M aqueous lithium hydroxide (5 mL, 10 mmol) was added to a stirred solution of methyl 2-chloro-4-(1-phenyl-5-(trifluoromethyl)-1*H*-pyrazole-4-carboxamido)benzoate (1.82 g, 3.2 mmol) in a mixture of tetrahydrofuran (5 mL) and methanol (5 mL) at room temperature. After stirring for 2 h the reaction was warmed to 35 °C for an additional 2 h. The mixture was concentrated under reduced pressure and the aqueous remains diluted with water. The pH was adjusted by the addition of aqueous 1 M hydrochloric acid to pH ~3 then extracted with ethyl acetate (x 2). The combined organic extracts were washed with water, saturated brine, dried over magnesium sulphate, filtered and concentrated. The crude product was dissolved in methanol, adsorbed onto diatomaceous earth and purified by silica gel chromatography (Isco Combiflash, 80 g; gradient elution: 15% ethyl acetate/hexane for 2 min then 15% to 100% ethyl acetate/hexane over 22 min at 60 mL/min) via detection with UV light at 254nm to afford 2-chloro-4-(1-phenyl-5-(trifluoromethyl)-1H-pyrazole-4-carboxamido)benzoic acid (0.929 g, 70%) as an off-white solid. m/z (ES+), [M+H]+ = 410.2; C18, 2–98% acetonitrile (0.1% aqueous formic acid), 2min, HPLC tR = 1.01 min. ^1^H NMR (300 MHz, DMSO-*d*_6_) δ ppm 7.50–7.57 (m, 2 H) 7.58–7.63 (m, 3 H) 7.71 (dd, *J* = 8.67, 2.07 Hz, 1 H) 7.88 (d, *J* = 8.48 Hz, 1 H) 7.97 (d, *J* = 2.07 Hz, 1 H) 8.34 (s, 1 H) 10.85 (s, 1 H) 13.14 (br s, 1 H).
Step 3
  *N*,*N*-Diisopropylethylamine (1.3 mL, 7.3 mmol) was added to a stirred solution of azetidin-3-ol hydrochloride (481 mg, 4.4 mmol) and 2-chloro-4-(1-phenyl-5-(trifluoromethyl)-1*H*-pyrazole-4-carboxamido)benzoic acid (600 mg, 1.5 mmol) in anhydrous dimethylformamide (10 mL) under nitrogen. To this was added 1-[bis(dimethylamino)methylene]-1*H*-1,2,3-triazolo[4,5-b]pyridinium 3-oxid hexafluorophosphate (HATU) (1.11 g, 2.9 mmol) and the resulting solution was allowed to stir at room temperature for 20 h. The solution was diluted with ethyl acetate and washed with saturated aqueous sodium hydrogen carbonate, saturated brine (x 2), dried over magnesium sulphate, filtered and concentrated. The crude product was taken up in methanol, adsorbed onto diatomaceous earth and purified by silica gel chromatography (Isco Combiflash, 20 g; gradient elution: 1% methanol/dichloromethane for 2 min then 1% to 7% methanol/dichloromethane over 16 min at 30 mL/min) via detection with UV light at 254 nm to afford a white solid. This was subjected to supercritical fluid chromatography (2-ethylpyridine 4.6 mm x 150 mm 5μm column; gradient elution:10–55% methanol in CO_2_ over 5 min at 5mL/min, 40 °C, 100 bar outlet pressure). After concentration, the resulting material was dried under vacuum overnight at 40 °C/~50 mTorr then again at 50 °C/~50 mTorr overnight then again at 60 °C/~35 mTorr overnight to afford *N*-(3-chloro-4-(3-hydroxyazetidine-1-carbonyl)phenyl)-1-phenyl-5-(trifluoromethyl)-1*H*-pyrazole-4-carboxamide (Compound 2) (408 mg, 60%) as a white solid. m/z (ES+), [M+H]+ = 465.2; C18, 2–98% acetonitrile (0.1% aqueous formic acid), 2min, HPLC tR = 0.89 min. m/z (ES+), [M+H]+ = 465.2; C18, 2–98% acetonitrile (0.1% aqueous NH_4_OH), 2min, HPLC tR = 0.86 min. ^19^F NMR (282 MHz, DMSO-*d*_6_) δ ppm -54.79. ^1^H NMR (300 MHz, DMSO-*d*_6_) δ ppm 3.66–3.83 (m, 2 H) 4.05 (t, *J* = 8.19 Hz, 1 H) 4.18–4.28 (m, 1 H) 4.46–4.58 (m, 1 H) 5.78 (d, *J* = 6.03 Hz, 1 H) 7.42 (d, *J* = 8.48 Hz, 1 H) 7.51–7.58 (m, 2 H) 7.59–7.64 (m, 3 H) 7.65–7.70 (m, 1 H) 7.94 (d, *J* = 2.07 Hz, 1 H) 8.34 (s, 1 H) 10.79 (s, 1 H).

### MALT1 FRET assay

The assay detected compounds that inhibited the MALT1 cleavage of the four amino acid (LRSR) FRET substrate resembling BCL10 protein, one of the natural occurring substrates for MALT1. The four amino acid peptides were labelled with the fluorophore Rhodamine 110 (Rh110) as a donor and a non-fluorescent quencher (Ac). When the peptide is cleaved by MALT1 after the second arginine (R), this results in an increase in donor signal. Inhibition of enzyme activity can be measured as absence of increase in signal over time. Sodium citrate was shown to add stability to the protein (DSF) and increased the FRET signal up to 0.67M. It was decided that the assay would be run at 0.5M sodium citrate since the assay signal was considered sufficient.

The Km for Ac-LRSR-Rh110 (peptide) was calculated to 13 and 20 μM in two separate experiments and the final assay concentration of 20 μM was chosen.

A final enzyme concentration of 1 nM was used with a reaction time of 3–4 h to get a good enough assay window. The reaction was found to be linear for at least 5 h.

Reagents were as follows: Hepes-NaOH pH 6.86 Sigma (H3375), EDTA Sigma (E7889), CHAPS, G Biosciences (DG096), TCEP Invitrogen (T2556), sodium citrate Merck (1.06448.1000), (Ac-Leu-Arg-Ser-Arg)-2Rh110 (Ac-LRSR-Rh110) AnaSpec (AS-PE90-2504). MALT1 protein was prepared in house (pbCPSS1800).

The assay buffer was prepared on the day of the experiment 0.025 mM HEPES pH 6.86, 0.1 mM EDTA, 0.05% CHAPS, 1 mM TCEP, 750 mM sodium citrate, and was used to prepare 2 nM MALT1 pbCPSS1800 and 40 mM peptide stock solutions.

The assay was run in Greiner medium bind black 384-well low volume microplate (#784076). The compounds were predispensed into these as assay ready plates from 10 mM stocks in DMSO to give 10-point dose response curves in 9 half-log dilution steps with top assay concentration of 50 mM and lowest assay concentration of 3.125 nM. DMSO was used as a negative control and mepazine was used at 5 mM as a positive inhibition control. To begin the assay 2 ml of enzyme solution was dispensed into the assay plate and allowed to stand for 30mins at room temperature. To each well was added 2 ml of 40 mM peptide solution. The assay plate spun at 800 rpm for 5 seconds to ensure no reagents were left stuck to the edge of the well. The plate was sealed and left for 3–4 hours at RT. The fluorescence of the wells was read on a PhHERAstar reader, excitation 485 nm emission 520 nm, flying mode, focal height 10.1 mm gain set 390. DMSO was used as a null control. The data was analysed in Genedata (https://www.genedata.com).

Calculation method:

Compound%effect=100*[(X-min)/(max-min)],

where X represents the normalized value for the compound based on the 0 and 100% controls.

### Secondary pharmacology

All secondary pharmacology assays (Table 1) were performed by Eurofins (www.eurofins.com).

**Table 1:** MALT1 potency and selectivity profile across 156 common pharmacological targets (human unless otherwise stated) of mepazine, and tool Compounds 2 and 3, (data from Eurofins [26]), n.t. = not tested, pIC50 = negative logarithm of the concentration of compound that produce 50% inhibition/antagonism of the named target, pEC50 = negative logarithm of the concentration of the named compound that produces half the maximal agonistic effect of the named target protein.

Target Assay	Mepazine	Cpd 2	Cpd 3
	Cpd 1		
MALT1 FRET pIC_50_	5.9	6.9	8.5
a1B pIC_50_	>8.0	<4.0	<4.0
H1 pIC_50_	>8.0	<4.0	<4.5
Sigma1 pIC_50_	>8.0	<4.0	<4.0
H1 Antagonist pIC_50_	7.9	4.5	n.t.
M5 pIC_50_	7.4	<4.0	<4.0
M1 IC_50_	7.4	<4.0	<4.0
a1A binding pIC_50_	7.3	<4.0	4.2
M1 Antagonist pIC_50_	7.0	4.8	n.t.
a1A Antagonist pIC_50_	6.9	4.3	<4.0
a1B Antagonist pIC_50_	6.8	<4.0	<4.5
M2 Binding pIC_50_	6.4	4.1	<4.0
D3 pIC_50_	6.2	<4.0	<4.0
a2C pIC_50_	6.1	<4.0	<4.0
5HT2B Binding pIC_50_	6.1	4.8	5.5
Ca2+ channel L-verapamil site Rat binding pIC_50_	6.1	<4.0	<4.0
NET Hu Bind pIC_50_	5.9	<4.0	<4.0
5-HT2C pIC_50_	5.9	<4.0	<4.0
5HT3 pIC_50_	5.8	<4.0	<4.0
5HT7 agonist pIC_50_	5.8	<4.0	<4.0
D2 pIC_50_	5.7	<4.0	<4.0
OPRk1 pIC_50_	5.7	<4.0	<4.0
M2 Antagonist pIC_50_	5.7	<4.0	<4.5
5-HT2C agonist pEC_50_	5.7	<4.0	<4.0
5HT1B Rat Bind pIC_50_	5.6	4.7	<4.0
SST4 Hu Bind pIC_50_	5.6	<4.0	<4.0
DOP2 Antagonist pIC_50_	5.3	<4.5	n.t.
AT pIC_50_	<4.0	5.3	4.8
A1 bind pIC_50_	<4.0	5.3	5.7
OPRm1 Hu Bind pIC_50_	5.2	<4.0	4.5
CaV-L pIC_50_	5.2	4.1	<4.0
A1 Agonist pEC_50_	5.2	<4.5	<4.5
EtA Agonist pEC_50_	5.1	<4.0	n.t.
5HT2B Antagonist pIC_50_	5.0	5.0	<4.0
D1 bind pIC_50_	5.0	4.0	<4.0
NaV1.5 pIC_50_	4.9	<4.5	<4.5
OPRk1 Agonist pEC_50_	4.9	<4.0	n.t.
A2A bind pIC_50_	4.9	<4.0	<4.0
A2A pIC_50_	<4.0	4.9	n.t.
OPRm1 Hu CHO cAMP TRF Agonist CR) pEC_50_	4.8	<4.0	<5.0
H2 pIC_50_	4.8	<4.0	<4.0
Ghre Agonist pEC_50_	4.7	<4.0	n.t.
NK1 pIC_50_	4.7	<4.0	<4.1
5-HT1D pIC_50_	4.7	<4.0	<4.0
NK1 Antagonist pIC_50_	4.7	4.2	n.t.
SST4 Agonist pEC_50_	4.6	<4.0	n.t.
β2 pIC_50_	4.6	<4.0	<4.0
D2 Antagonist pIC_50_	4.6	<4.5	n.t.
DAT bind pIC_50_	4.6	<4.0	<4.0
A2C Antagonist pIC_50_	4.5	<4.0	n.t.
M1 Agonist pEC_50_ DOP2 pIC_50_	4.5	<4.0	n.t.
A2A Antagonist pIC_50_	4.5	<4.5	<4.5
5-HT1A pIC_50_	4.5	<4.0	<4.0
Kv4.3 pIC_50_	4.1	4.5	<4.5
Ang2 AT1 pIC_50_	<5.0	4.5	n.t.
5HT4 pIC_50_	4.3	4.5	<4.0
5-HT7 Antagonist pIC_50_	4.4	<4.0	<4.0
Ang2 AT1 Agonist pEC_50_	4.4	<4.0	n.t.
Ghre pIC_50_	4.4	4.0	<4.6
MAO-B pIC_50_	4.4	<4.0	n.t.
MR2 pIC_50_	4.4	<4.0	<4.0
SET pIC_50_	4.4	4.0	<4.0
5-HT2C Antagonist pIC_50_	<4.0	4.3	<4.0
NAchA7 Bind pIC_50_	4.3	<4.0	<4.0
A2C Agonist pEC_50_	4.3	<4.0	n.t.
Hu HepG2 Tox pGlucose IC_50_	4.3	<3.6	<3.6
THP1 pIC_50_ Cytotox	4.3	<3.6	<3.6
GR pIC_50_	4.3	<4.0	<4.0
NK1 Agonist pEC_50_	4.3	<4.0	n.t.
β1 pIC_50_	4.3	<4.0	<4.5
PPARγ pIC_50_	4.2	<4.0	<4.0
BK2 Agonist pEC_50_	4.2	<4.0	n.t.
CB1 bind pIC_50_	4.2	<4.0	<4.0
NMDA pcp binding site rat binding pIC_50_ a1A Agonist pEC_50_ TRKA pIC_50_β1 bind pIC_50_	4.1	<4.0	<4.0
D3 Agonist pEC_50_	<4.0	4.1	<4.0
MAP3K7 pIC_50_	4.0	4.0	4.0
Hu HepG2 Tox Lumin pGalactose IC_50_	4.0	3.7	<3.6
H1 Agonist pEC_50_ COX1 pIC_50_	4.0	<4.0	n.t.
Choride ion channel–GABA gated Rat Bind pIC_50_	<4.0	4.0	5.5
Ghrehlin Antagonist pIC_50_	<5.5	<5.5	n.t.
BK2 Antagonist pIC_50_	<5.5	<4.0	n.t.
5-HT1D Agonist pEC_50_ 5-HT1D Antagonist pIC_50_ D2 Agonist GABAB Agonist pEC_50_ GABAB Antagonist pIC_50_pEC_50_ DOP2 Agonist pEC_50_5-HT1A Agonist pEC_50_5-HT1A Antagonist pIC_50_	<4.5	<4.5	n.t.
β2 Antagonist pIC_50_	<4.5	<4.0	n.t.
TXA2 pIC_50_	<4.0	4.2	<4.0
ACHE pIC_50_	<4.0	<4.0	<4.5
A2A Agonist pEC_50_ 5HT1B Agonist pEC_50_	<4.5	<4.5	<4.5
β1 Agonist pEC_50_ M2 Agonist pEC_50_	<4.0	<4.0	<5.0
5HT1B Antagonist pIC_50_	<4.5	<4.5	<5.5
IKs pIC_50_	<3.8	<4.5	<4.5
CB1 Agonist pEC_50_ CB1 Antagonist pIC_50_ D1 Agonist pEC_50_ D1 antagonist pIC_50_ OPRm1 Hu CHO cAMP TRF Antagonist CR) pIC_50_ MMP2 pIC_50_	<4.0	<4.0	<4.5
ECE1 pIC_50_ COX2 pIC_50_ SST4 Antagonist pIC_50_ MR2 Agonist pEC_50_ MR2 Antagonist pIC_50_ eNOS pIC_50_ OPRk1 Antagonist pIC_50_β2 Agonist pEC_50_ A2A Agonist pEC_50_ A2A Antagonist pIC_50_ PI3Kα pIC_50_5HT4 Agonist pEC_50_; 5HT4 Antagonist pIC_50_	<4.0	<4.0	n.t.
ALK4 pIC_50_; Ang2 AT1 pIC_50_; CatS Hu pIC_50_; NAchA4 Hu Bind) pIC_50;_ NAchA1 Hu Bind pIC_50_; H2 Hu CHO cAMP TRF Antagonist CR pIC_50_;5HT2B Agonist pEC_50_; ROCK2 pIC_50_ KDR pIC_50_; INSR pIC_50_; Central GABA benzodiazepine receptor Rat Bind pIC_50_; ROCK1 pIC_50_; Src pIC_50_ TSPO Hu pIC_50_; PDK1 pIC_50_; Glycine receptor strychnine sensitive agonist site rat binding pIC_50_; NMDA Rat Bind pIC_50_;RARα pIC_50_ MAP3K7 pIC_50_; GSK3β pIC_50_; BK2 pIC_50;_ H2 Agonist pEC_50_; EtA pIC_50_;AurKA pIC_50_; D3 Antagonist pIC_50_; PDE6 pIC_50_; FGFR pIC_50_; CatS pIC_50_; cKIT pIC_50_ Na+/K+ ATPase pIC_50_; GABA α1β2γ2 pIC_50;_ 5-HT7 Agonist pEC_50_; a1B Agonist pEC_50_; EGFRK pIC_50_	<4.0	<4.0	<4.0

### Blood collection, isolation of PBMCs and isolation of human cell subsets

Healthy donors were recruited from AZ volunteers and all samples were taken following appropriate blood collection guidelines. All blood donor volunteers signed Informed Consent form and donation was approved by AstraZeneca Institutional review board and local Ethic committee (033–10). PBMCs were isolated from the blood by density-gradient centrifugation in Leucosep tubes (Greiner Bio-One) filled with Ficoll-Paque plus (GE Healthcare) or using LymphoPrep density gradient medium and SepMate tubes (Stem Cell Technologies) for the Dendritic cell work. All cells were purified by either negative or positive selection using the appropriate isolation kits (see below) according to the manufacturer’s instructions (Miltenyi Biotech).

### Stimulation of myeloid cells

Purified PBMCs were resuspended in Iscove´s Modified Dulbecco´s Medium (Gibco) + 10% fetal bovine serum (Gibco) and z-VRPR-fmk (100 μM), mepazine (10 μM) or Compound 2 (10 μM) were added. After 30 minutes, cells were stimulated either with 100 μg/mL zymosan (Zymosan-depleted, InvivoGen) or 20 ng/mL LPS (from E-coli, InvivoGen) for 5 hours. BD GolgiStop (BD Biosciences) was added 1 hour after LPS or zymosan addition. Following stimulation, cells were analysed by flow cytometry for cell surface markers and intracellular cytokines on a BD LSR Fortessa.

In some experiments, myeloid dendritic cells were separated from PBMCs using EasySep Human Myeloid DC Enrichment Kit (Stem Cells, #19061). CD14+ Monocytes were isolated using anti-CD14 MicroBeads (Miltenyi, #130-050-201). CD16-expressing monocytes were further purified from the CD14-negative fraction using anti-CD16-PE (BD Biosciences, #555407) and anti-PE microbeads (Miltenyi, #130-048-801). The CD16 negative fraction constituted the CD14+ cells(CD14+/CD16-). Purified mDCs or monocytes were stimulated overnight with 10 ng/mL LPS (from E-coli, InvivoGen) and 50 μg/mL zymosan (InvivoGen). The supernatants from mDCs (4x10^4^-13x10^4^ cells), CD14+ monocytes (7x10^4^-14x10^4^ cells) or CD16+ monocytes (3x10^4^-5x10^4^ cells), were analysed for secreted levels of IL-1β, IL-23, IL-12p70, IL-6, TNF-α and IL-10, using a custom-made multiplex (U-plex) ELISA assay (Meso Scale Discovery) according to the manufacturer’s instructions. Output values were normalized to cell number and statistical significance between treatments was estimated using one-way ANOVA with Dunnet’s post test.

### Stimulation of purified human CD4+ T cells

Human naive CD4+CD45RA+ cells were isolated from PBMC using a naïve CD4+ T cell Isolation kit (Miltenyi, #130-094-131) and stained with 0.25 μM cell trace violet (In vitrogen) and plated out (1x10^5^ cells/well) into a 96-well flat bottom plates (Nunc) coated with 1 μg/mL anti-CD3 antibody (OKT3-eBioscience). Soluble anti-CD28 (CD28.2-eBioscience) was added at a final concentration of 5 μg/mL and cells were cultured for 3 days.

For the tetanus toxoid antigen specific assay, monocytes were derived into Mo-DCs with 100 ng/mL GM-CSF and 40 ng/mL IL-4 (both PeproTech) as described elsewhere for 5 to 6 days. Mo-DCs were pulsed with 2.5 μg/mL tetanus Toxoid (Calbiochem) and matured with 100 ng/mL LPS (SIGMA-Aldrich) for 24 h. Mo-DCs were washed and co-cultured at a 1:50 ratio with CD4+CD45RO+ purified cells stained with 0.25 μM cell trace violet (Invitrogen) in a 96-well flat bottom plates (Nunc). Cells were incubated for 6 days.

In some experiments, human CD4+ memory T cells were cultured with autologous monocytes at a ratio 10:1 in the presence of soluble anti-CD3 and anti-CD28 antibodies (both eBioscience) and 100 ng/mL LPS (SIGMA-Aldrich) for 5 days in a 96-well flat bottom plates (Nunc).

In all experiments, cells were cultured in RPMI 1640 supplemented with 10% heat-inactivated FCS (Gibco) in triplicate. All cultures were incubated at 37 °C 5% CO_2_. In all experiments, Compound 2 was used at 10 μM, mepazine at 5 μM and z-VRPR-fmk a peptide inhibitor of MALT1 proteolytic activity at 100 μM unless otherwise indicated.

### Stimulation of purified human B cells

Human B cells were stained with 0.25 μM CFSE (Invitrogen) and plated out into a 96-well V-bottom plate (5x10^4^ cells/well) in triplicate. Cells were stimulated for 5 days with 5 μg/mL anti-IgM (Jackson ImmunoResearch), 100 ng/mL anti-CD40 (R&D) and 10 ng/mL IL-21 (PeproTech) final concentration.

For the B cell co-culture with CD4+CD45RO+CXCR5+ T cells, CD4+ T cells were first enriched by positive selection using CD4 microbeads (Miltenyi Biotech), then stained with CD45RO (BD) and CXCR5 (Biolegend) antibodies in PBS-0.5% BSA-2 mM EDTA. CD4+CD45RO+CXCR5+ were FACS sorted on an ARIA-III (BD Bioscience).

CFSE stained B cells and CD4+CD45RO+CXCR5+ T cells were plated out into a 96-well V-bottom plate at a 1:1 ratio and cells were stimulated with SEB 10 ng/mL (SIGMA-Aldrich), IL-21 10 ng/mL and BAFF 10 ng/mL (PeproTech) final concentration. Cells were incubated for 5 days.

Supernatants were collected and antibody production was analysed using a multiplex isotyping Panel 1 kit (Meso Scale Discovery).

### Treg induction, expansion and suppression assay

For Treg induction, naïve CD4+ T cells (1.5 x 10^5^ cells/well) were plated out in a 96-well flat bottom plates (Nunc) and stimulated with anti-CD3+ anti-CD28 antibody-coated Dynabeads (life Technologies) at a ratio 1:2 in the presence of 5 ng/mL TGF-β, 10 ng/mL IL-2 (both PeproTech) and 100 nM rapamycin (SIGMA-Aldrich). In all experiments, Compound 2 was used at 10 μM, mepazine at 5 μM and z-VRPR-fmk at 100 μM. Cells were incubated for 5 days at 37 °C 5% CO_2_. Dynabeads were then removed and cells were transferred to a 96-well round bottom plate. Cells were washed and incubated for another 2 days with 10 ng/mL IL-2 only before flow cytometric analysis.

For expansion experiments, naïve Tregs were isolated using CD4+CD25+CD45RA+ (Miltenyi Biotech, #130-093-631). Cells were distributed in a 96-well round bottom plate (Nunc) and stimulated with anti-CD3+anti-CD28 antibody coated expander Dynabeads (Life Technologies) according to the manufacturer’s instructions. Rapamycin (100 nM) was added to avoid loss of FOXP3 expression during the expansion phase. IL-2 and rapamycin were replenished every 2 days.

After 7 days expansion, cells were washed and transferred in a 48-well culture plate (Nunc). Cells were cultured in presence of 10 ng/mL IL-2 for an additional 4 days before cells were restimulated with anti-CD3+anti-CD28 antibody coated activator Dynabeads at a ratio 1:2 in the presence of 10 μM Compound 2, 5 μM mepazine, 100 μM z-VRPR-fmk or 100 nM rapamycin. Cells were incubated for 3 days at 37 °C 5% CO_2_ prior setting-up the suppression assay.

For the suppression assay, expanded Tregs were washed and then mixed with cell trace violet stained autologous naïve CD4+ responder cells in a ratio 1:1, 1:2 or 1:4 in the presence of autologous mitomycin-C (SIGMA-Aldrich) treated PBMCs and 200 ng/mL soluble anti-CD3 (OKT3. eBioscience). Cells were incubated for 3 days at 37 °C 5% CO_2_ and proliferation of the responder cells was analysed by flow cytometry on a BD Canto II.

### Flow cytometry and intracellular staining

Antibody combinations were used to identify and quantify specific cell types in cultures. Fluorochrome-conjugated mAbs to human CD3 (BV421; Alexa 488), CD4 (PerCp-Cy5.5; Horizon V450 and APC-H7), CD25 (FITC; APC), CD45RO (APC-H7), CD45RA (BV510), FoxP3 (PE), IFN-ɣ (Alexa 647), IL-17A (PE), IL-2 (BV510), CD27 (BV421), CD56 (Alexa Fluor 488), HLA-DR (BV786), CD123 (BV711), CD80 (BUV395), CD86 (Alexa Fluor 700), CD83 (APC), CD19 (PerCp-Cy5.5; Alexa 488), IgD (PE-cy7), IgG (BV510) and CD38 (APC) were purchased from BD Bioscience. CXCR5 (PE), BDCA-2 (PE), CD1c (PerCp-Cy5.5), CD11c (BV421) and CD14 (BV605) were purchased from Biolegend. pIκBα (eFluor660) and pErk (PerCp-eFluor710) were obtained from eBioscience.

Cell viability was analysed by staining the cells with Fixable Viability Dye eFluor 780 purchased from eBioscience or LIVE/DEAD fixable aqua stain (Molecular probes).

For intracellular cytokine staining in human CD4+ T cells, cells were incubated for 4 h at 37 °C 5% CO_2_ in the presence of 500 ng/mL ionomycin, 10 ng/mL PMA and 10 μg/mL Brefeldin A (all obtained from SIGMA-Aldrich). Cells were washed in PBS, stained with the Fixable Viability Dye (eBioscience) and cell surface receptor antibodies for 20 min at 4 °C. Cells were washed twice with PBS-0.5% BSA-0.05% NaN_3_ then fixed with BD Fixation/permeabilisation buffer for 15 min at 4 °C. After washing the cells in the BD permeabilisation buffer, cells were stained with IFN-ɣ, IL-2 and IL-17A (when appropriate) for 30 min at 4 °C. Cells were washed twice with the BD permeabilisation buffer and resuspended in PBS-0.5% BSA-0.05% NaN_3_ for analysis.

For expression of intracellular cytokines in myeloid cells after LPS or Zymosan stimulation, PBMCs were stained with LIVE/DEAD fixable aqua stain (Molecular Probes) and for cell surface markers with human antibodies against CD3 (Alexa Fluor 488), CD19 (Alexa Fluor 488), CD56 (Alexa Fluor 488), HLA-DR (BV786), CD123 (BV711), CD80 (BUV395), CD86 (Alexa Fluor 700), CD83 (APC), BDCA-2 (PE), CD1c (PerCpCy5.5), CD11c (BV421) and CD14 (BV605). Cells were fixed in 4% paraformaldehyde, permeabilized using the BD Cytofix/Cytoperm buffer (BD Biosciences) and stained with anti-TNF-α (BUV395) and anti-IL-6 (APC) (BD Biosciences).

For FoxP3 intracellular staining, cells stained with Fixable viability Dye eFluor 780 and for cell surface CD4 and CD25 for 20 min in PBS, washed in PBS-0.5% BSA-0.05% NaN_3_ and fixed with the FoxP3 fixation buffer (eBioscience) for 30 min at 4 °C. Cells were then washed with the FoxP3 permeabilisation buffer (eBioscience) and stained with anti-FoxP3 antibodies for 45 min at 4 °C. Cells were washed twice in permeabilisation buffer and resuspended in PBS-0.5% BSA-0.05% NaN_3_ for analysis.

For phosphorylation analysis, PBMCs were incubated O/N with Compound 2, mepazine or z-VRPR-fmk at indicated concentrations in 96 well-round bottom plate. Cells were then washed in RPMI and resuspended in 50 μL RPMI1640 containing 50 ng/mL PMA and 500 ng/mL ionomycin for 10 min at 37 °C. Cells were then immediately fixed with 150 μL paraformaldehyde 2% solution in PBS (affymetrix). Cells were centrifuged and resuspended in Fix/perm Solution (BD bioscience stained with CD4, CD45RA and CD19 (BD). After permeabilisation cells were stained with phospho-IκBα and phospho-ERK (eBioscience).

Flow cytometric data were acquired on a FACS Canto II or an LSR Fortessa (BD Bioscience) and the data was analyzed with FlowJo software.

### Immunoblotting

Purified Human CD4+ T cells (Miltenyi Biotech) were pre-incubated with 10 μM proteasome inhibitor MG132 (SIGMA-Aldrich) and 10 μM Compound 2, 10 μM mepazine or 300 μM z-VRPR-fmk for 30 min prior stimulation with 10 ng/mL PMA and 250 ng/mL ionomycin. After 1 h stimulation, cells were lysed in RIPA buffer and protease inhibitor (cOmplete; Roche). Proteins were separated by SDS-polyacrylamide gel electrophoresis and electrotransferred to nitrocellulose. Membrane was blocked with 5% bovine serum albumin and probed with anti-CYLD (D1A10; Cell Signaling Technology) or anti-RELB (C1E4; Cell Signaling Technology). After incubation with IRDye conjugated secondary antibodies (LI-COR), proteins were detected using a Odyssey imager (LI-COR).

### Animals

Female C57BL/6 mice (8 weeks, Charles River laboratories, UK) were group housed in pathogen-free conditions in a temperature-controlled environment (20–21 °C) with a relative humidity of 55±15% and a light-dark cycle of 12 h (lights on at 06:00 and lights off at 18:00). They were provided food (R70, Lantmännen, Sweden) and water *ad libitum*.

Animal experiments were approved by the Local Ethical committee in Gothenburg (123–2014).

### Analysis of MALT1 inhibition in vivo

For analysis of the effect of MALT1 inhibition on cytokine release in vivo, C57BL/6 mice were injected I.P with Compound 2 at 30 or 90 mg/Kg or vehicle 30 min prior anti-CD3 antibody (145-2C11. BD) or IgG1κ isotype control (A19-3. BD) injection (I.P). 4 hours after anti-CD3 antibody or isotype injection, mice were sacrificed and blood was collected in EDTA tubes (microvette 600) for cytokine analysis using V-PLEX proinflammatory Panel 1 kit (Meso Scale Discovery). For the 4 week safety study, C57BL/6 mice were dosed orally twice a day with 10 mg/Kg Compound 3. After 4 weeks, mice were euthanized by inhalation of isoflurane and exsanguination. Blood was collected in EDTA tubes (microvette 600) for cytokine analysis using V-PLEX proinflammatory Panel 1 kit (Meso Scale Discovery). Lymph nodes and spleen were collected and mashed. Cells were analysed by multicolor flow cytometry and numbers of IFN-γ producing cells or FoxP3 regulatory T cells were calculated.

### Histopathology

Mice were necropsied at 12 weeks of age and tissues were sampled and weighed, including adrenal glands, brain, heart, kidney, liver, lung, mesenterial lymph nodes, pancreas, spleen, thymus, sublingual gland, parotis, stomach, duodenum, jejunum, ileum, cecum, and colon. Tissues were subsequently fixated in paraffin, sectioned at 4–6 μm, stained with hematoxylin and eosin (H&E) for histopathological examination under microscope.

### RNA isolation and reverse transcriptase quantitative PCR (RT-qPCR)

Human CD4+ T cells were isolated by negative selection (Miltenyi Biotech) and plated out (2x10^5^ cells/well) into a 96-well flat bottom plates (Nunc) coated with 1 μg/mL anti-CD3 antibody (OKT3-eBioscience) in triplicate. Soluble anti-CD28 (CD28.2-eBioscience) was added at a final concentration of 5 μg/mL and cells were cultured for 3 days. Cells were then pooled, washed in PBS and total RNA was extracted using the RNeasy Plus Mini kit (Qiagen), according to the manufacturer’s instructions. Equal amount of RNA was reverse-transcribed using the High capacity RNA-to-cDNA Kit (Life Technologies). Quantitative PCR was conducted with QuantStudio 7 Flex real-time PCR system (Applied Biosystems) using the following TaqMan assays: INFG Hs00989291, GUSB Hs00939627, ACTB Hs01060665. Reactions were run in triplicate and Cycle thresholds (Ct values) were normalized to those of housekeeping genes GUSB and ACTB. Relative quantity calculations were performed using the 2-ddCt method using the DMSO-treated cells as reference sample.

## Results

### An allosteric MALT1 inhibitor modulates mainly protease function

**Fig 1:**
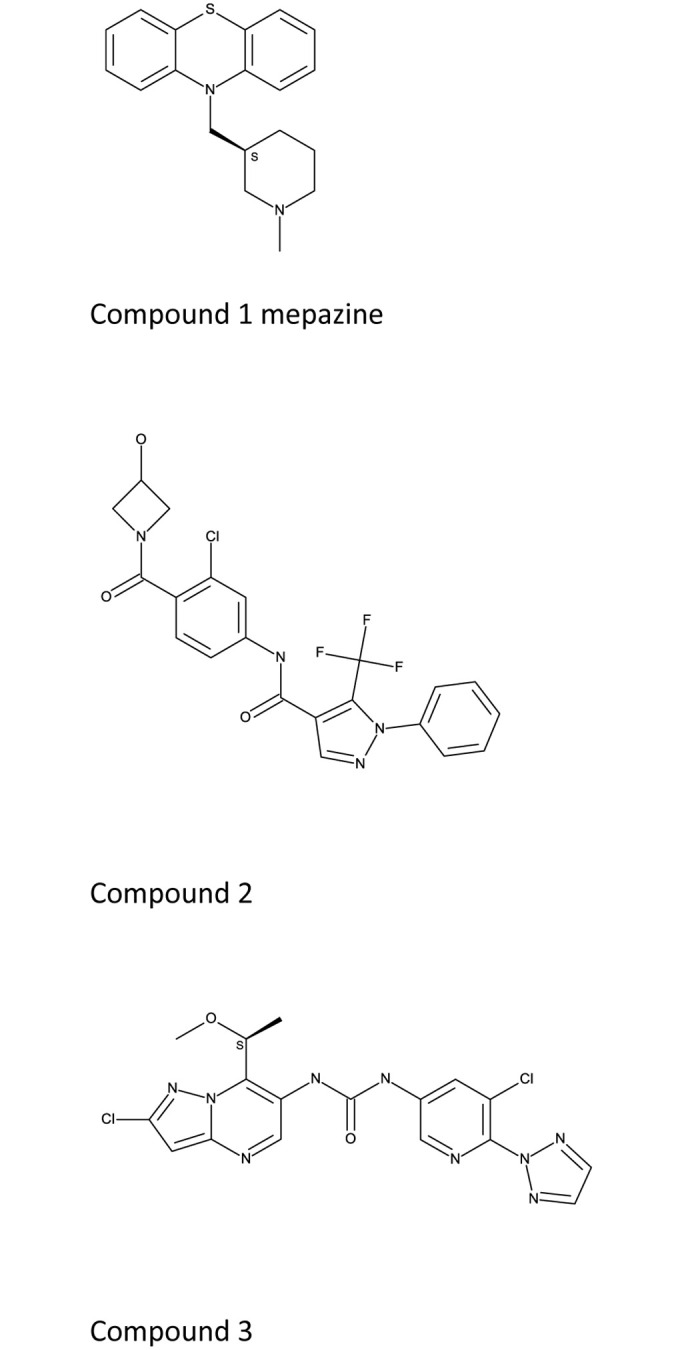
Structures of mepazine, compounds 2, 3.

Dissecting the role of pharmacological inhibition of MALT1 activity requires potent and selective chemistry tools. A MALT1 inhibitor, mepazine, and Fig 1 [26,27], and a closely related phenothiazine compound thioridazine, have been used as a MALT1 tool compounds *in vitro* and *in vivo*, and have been shown to bind at an allosteric site on MALT1 [27]. Mepazine suffers from low potency, off-target effects and cytotoxic effects [27,28]. Its selectivity profile makes it unsuitable tool to test the role of MALT1 in *in vitro* or *in vivo* studies (Table 1). The peptidic active site inhibitor, z-VRPR-fmk has been shown to also bind to cathepsins [29,30]. We therefore used as Compound 2 as tool compound, an allosteric MALT1 inhibitor (Table 1) originating from a drug discovery program at AstraZeneca. This compound binds at the same allosteric site as mepazine but is a more potent and selective MALT1 inhibitor (Table 1) but its high MALT1 potency and improved selectivity profile made it a very suitable tool to probe the role of MALT1 allosteric inhibition in cellular assays Compound 3 also binds at the same allosteric site as mepazine and Compound 2. Compound 3 is also a potent and selective MALT1 inhibitor, but it’s improved pharmacokinetic profile, and improved potency made it the preferred choice for in vivo experiments.

**Fig 2:**
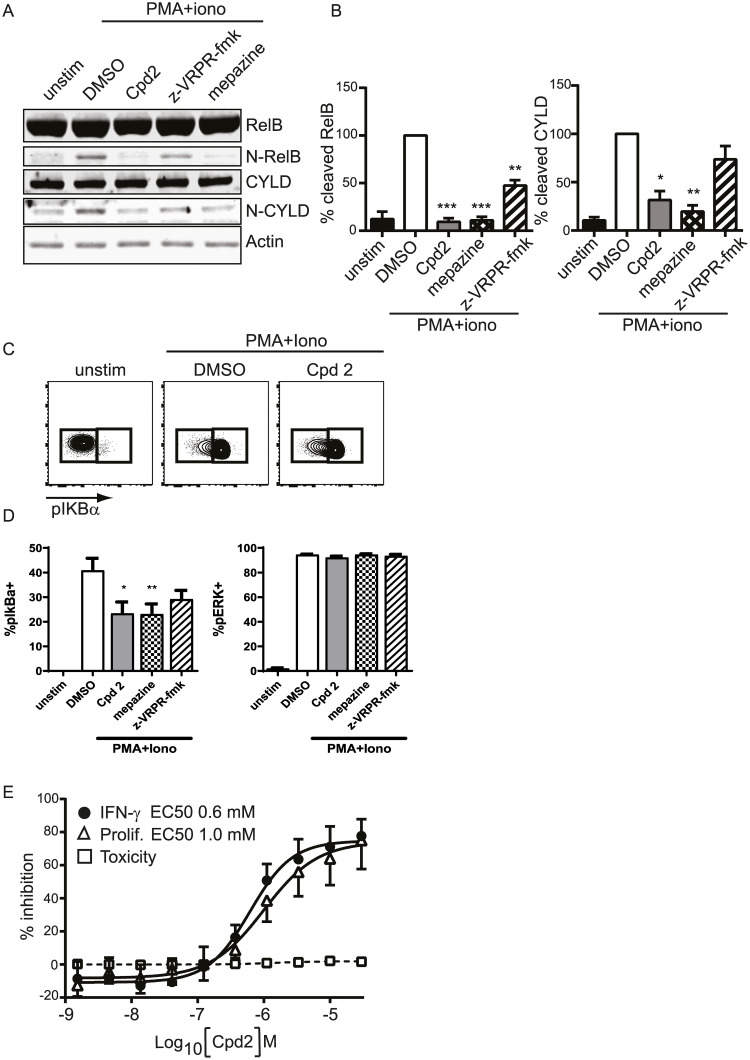
Characterization of allosteric MALT1 inhibitor Compound 2. Purified human primary CD4+ T cells were stimulated with PMA and ionomycin in the presence of MALT1 inhibitors. Compound 2 and mepazine were used at 10 μM and z-VRPR-fmk at 300 μM (A-B) or 100 μM (C-D). (A) Western Blot analysis of RELB and CYLD, their cleaved product (N-RELB and N-CYLD). (B) Quantification of cleaved RELB and CYLD. All quantitative data were normalised to actin signal and calculated as a percentage relative to DMSO signal set at 100%. Data are presented as mean+/SEM with n = 4. *:p<0.05; **:p<0.005; ***:p<0.001. (donor-matched one-way ANOVA with Dunnett’s multiple comparison test) (C) Representative FACS plots of phosphorylated IκBα in CD4+ T cells following stimulation with PMA and ionomycin for 10minutes. (D) Quantification of FACS analysis of pIκBα and pERK positive cells. Data are presented as mean+/SEM with n = 3. *p<0.05; **:p<0.005. (donor-matched one-way ANOVA with Dunnett’s multiple comparison test) (E) Inhibition of IFN-γ secretion (black circles) by Compound 2 in human CD4+ cells stimulated with anti-CD3 antibody plus anti-CD28 antibody for 3 days. Proliferation (open triangles) and cytotoxicity (open squares) were measured using CFSE and 7-AAD by flow cytometry 3 days post-stimulation. Data are shown as % inhibition from 6 donors (mean ± 95% CI). EC_50_ values are annotated.

First, we evaluated the effect of MALT1 inhibitors on scaffold and protease activity in PMA+ionomycin stimulated CD4+ T-cells. The cleavage of RELB was inhibited by allosteric inhibitors, Compound 2 and mepazine, and by the active site inhibitor z-VRPR-fmk (Fig 2A and 2B). The cleavage of CYLD was also inhibited by allosteric inhibitors Compound 2 and mepazine, but the inhibition did not reach significance for the active site inhibitor z-VRPR-fmk. We demonstrated a modest effect on the inhibition of the IKK-dependent phosphorylation of Iα with the allosteric MALT1 inhibitors, mepazine and Compound 2 in human CD4 T-cells, while the active site inhibitor did not reach statistical significance (Fig 2C and 2D). No inhibition of the phosphorylation of ERK was observed by MALT1 inhibitors indicating specific effect of MALT1 inhibition on NF-κB activation (Fig 2D). Furthermore, Compound 2 did not show toxicity at concentrations showing efficient inhibition of IFN-γ production and proliferation in activated CD4 T-cells (Fig 2E).

We observed a difference in the effect of allosteric vs active site inhibitors on phosphorylation of IκBα, which suggest that an allosteric inhibitor modulates both protease and, at least modestly, scaffold function of MALT1, the latter linked to IKK activation. Since total IκB decreases following T cell activation [31,32] and the p-IκB signal significantly increases in activated vs unstimulated cells it is unlikely (though not impossible) that the change in p-IκB signal is related to the change in total IκB levels.

Accordingly, the immunomodulatory effects of the allosteric inhibitor may differ from that of pure active site inhibitor.

### MALT1 inhibition decreases Dectin-1 driven cytokine production in human myeloid DCs

**Fig 3:**
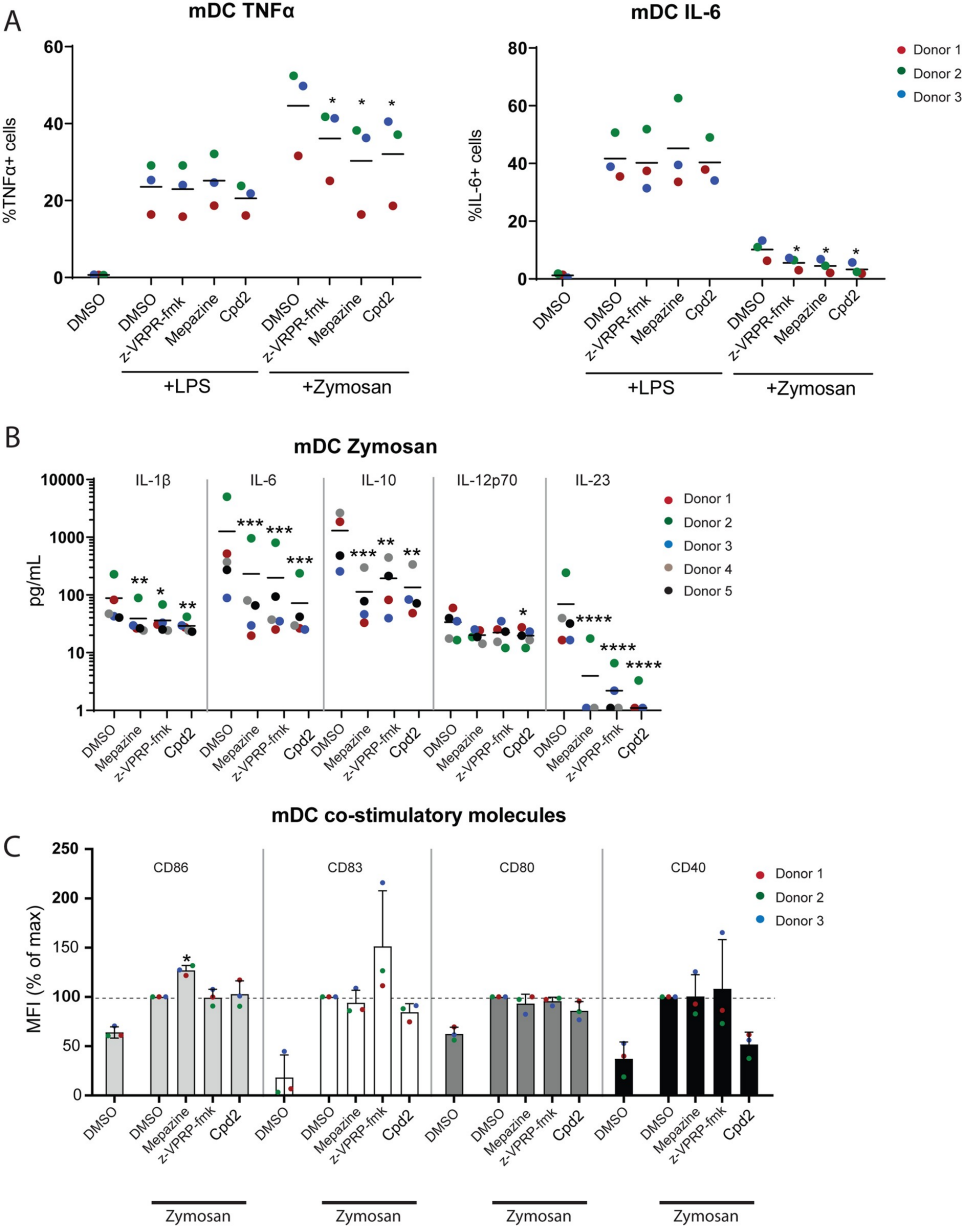
MALT1 inhibition in mDCs decreases cytokine production in zymosan-treated cells. (A) Unstimulated PBMCs, 5-hour LPS- or zymosan-stimulated PBMCs (pre-treated for 30 minutes with DMSO, 10 μM mepazine, 100 μM z-VRPR-fmk or 10 μM Compound 2) were analyzed by flow cytometry for intracellular expression of TNF-α or IL-6 in mDCs. Data is representative for 1 out of 3 independent experiments. Coloured dots indicate data from 3 individual donors. * p<0.05. (donor-matched one-way ANOVA with Dunnett’s multiple comparison test) (B) Expression of cytokines in the supernatant of *ex vivo* purified zymosan-treated mDCs after MALT1 inhibition as measured by MSD. Coloured dots indicate data from 5 individual donors. *:p<0.05; **:p<0.005; ***:p<0.001; ****:p<0.0001. (donor-matched one-way ANOVA with Dunnett’s multiple comparison test) (C) Flow cytometric analysis of the effect of MALT1 inhibition on the expression CD80, CD83, CD86 and CD40 on mDCs upon 5-hour zymosan stimulation of PBMCs. Bars represent mean ± SD and dots show individual donors (n = 3). In all experiments, values are normalized to zymosan-stimulated cells without MALT1 inhibition (set to 100%).

To investigate the effects of MALT1 inhibition on dendritic cells (DC) and monocyte activation and maturation, we stimulated peripheral blood mononuclear cells (PBMCs) or isolated myeloid DCs (mDCs) with zymosan, which binds to Dectin-1, an ITAM-containing receptor that signals via MALT1 [33]. The flow cytometry gating for the identification of human mDC and monocyte populations in PBMCs is shown in S2 Fig. Both MALT1 active site and allosteric inhibitors reduced the expression of IL-6 and TNF-α in zymosan-activated mDCs (Fig 3A). This effect was not seen when cells were stimulated with LPS, which signals to NF-κB through a MALT1-independent pathway (Fig 3A). Both MALT1 active site and allosteric inhibition almost completely abolished the secretion of IL-23 and IL-6 and reduced the secretion of IL-1β, IL-10 and IL-12p70 (Fig 3B) in purified human mDCs stimulated with zymosan but not LPS-treated mDCs. In *ex vivo* human CD14+/CD16- and CD14+/CD16+ monocytes activated *in vitro* with zymosan, a similar effect on cytokine release was observed, although the inhibition was primarily seen in cells treated with Compound 2 (S2 Fig). Again, no effect of MALT1 inhibitors on LPS-stimulated mDCs or monocytes was observed (S2 Fig).

Despite the altered cytokine expression observed upon MALT1 inhibition, the expression of activation markers CD80, CD83, CD86 and CD40 were unaltered by MALT1 inhibition in zymosan- or LPS-activated human mDCs (Fig 3C). This data suggests that MALT1 is necessary for efficient cytokine production and release but is dispensable for dendritic cell maturation upon Dectin-1 stimulation.

### A MALT1 allosteric inhibitor down-regulates Th1 and Th17 responses

**Fig 4:**
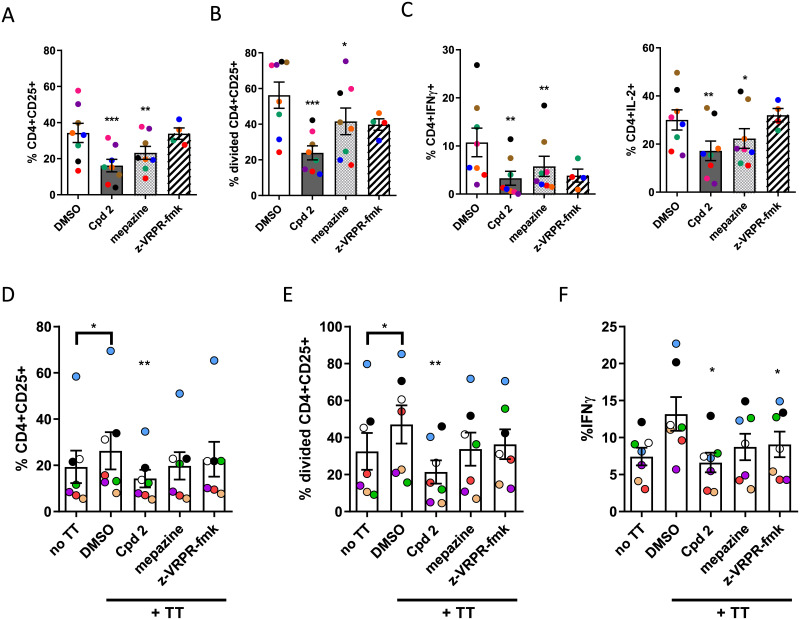
Effect of allosteric MALT1 inhibitors on activation, proliferation and cytokine production of human CD4+ T cells. The top panel (A-C) shows the effect of MALT1 inhibitors on CD4+CD45RA+ human T cells stimulated for 3 days with anti-CD3 + anti-CD28 antibody stimulated. Data is presented as the percentage of (A) cells expressing the activation marker CD25, (B) the percentage of cells that have divided, measured by Cell Trace Violet fluorescence intensity dilution and (C) the percentage of CD4+ cells producing IL-2 and IFN-ɣ analysed by multi-color flow cytometric analysis. Data are presented as mean ± SEM with n = 8. Coloured dots indicate data from individual donors across different stimulations.*:p<0.05; **:p<0.005; ***:p<0.001 (donor-matched one-way ANOVA with Dunnet’s multiple comparison test compared to DMSO control). The lower Panel shows the effect of the allosteric MALT1 inhibitor Compound 2 on human CD4+ CD45RO+ T cells co-cultured for 6 days with tetanus toxoid pulsed monocyte-derived dendritic cells in presence or absence of MALT1 inhibitors. Data is presented as the percentage of (D) cells expressing CD25, (E)the percentage of cells that have divided and (F) the percentage of cells producing IFN-ɣ as measured by flow cytometry. Experiments were considered positive when the mean proliferation of tetanus toxoid stimulated T-cells was greater than the mean+2SD of T-cells stimulated with unpulsed DCs. Data are presented as mean ± SEM with n = 7. Coloured dots indicate data from individual donors across different stimulations.*:p<0.05; **:p<0.001 (donor-matched one-way ANOVA with Dunnett’s multiple comparison test compared to DMSO control). In all experiments, 10 μM Compound 2, 5 μM mepazine or 100 μM z-VRPR-fmk were used.

Next, we evaluated the effect of MALT1 inhibition separately on naive and memory human CD4+ cells, previously depleted of CD25-expressing Tregs. We stimulated either CD4+CD45RA+ naive T cells with soluble anti-CD3 + anti-CD28 antibodies or activated CD45RO+CD4+ memory CD4 T cells with monocyte derived dendritic cells pulsed with a recall antigen, namely tetanus toxoid. In both cases, the percentage of CD25+ cells, the proliferation of CD25 positive cells and the percentage of IFN-γ producing CD4+ cells was inhibited by Compound 2 (Fig 4A–4F). We did not observe activation of IL-17 in memory CD4+ cells after tetanus toxoid stimulation.

**Fig 5:**
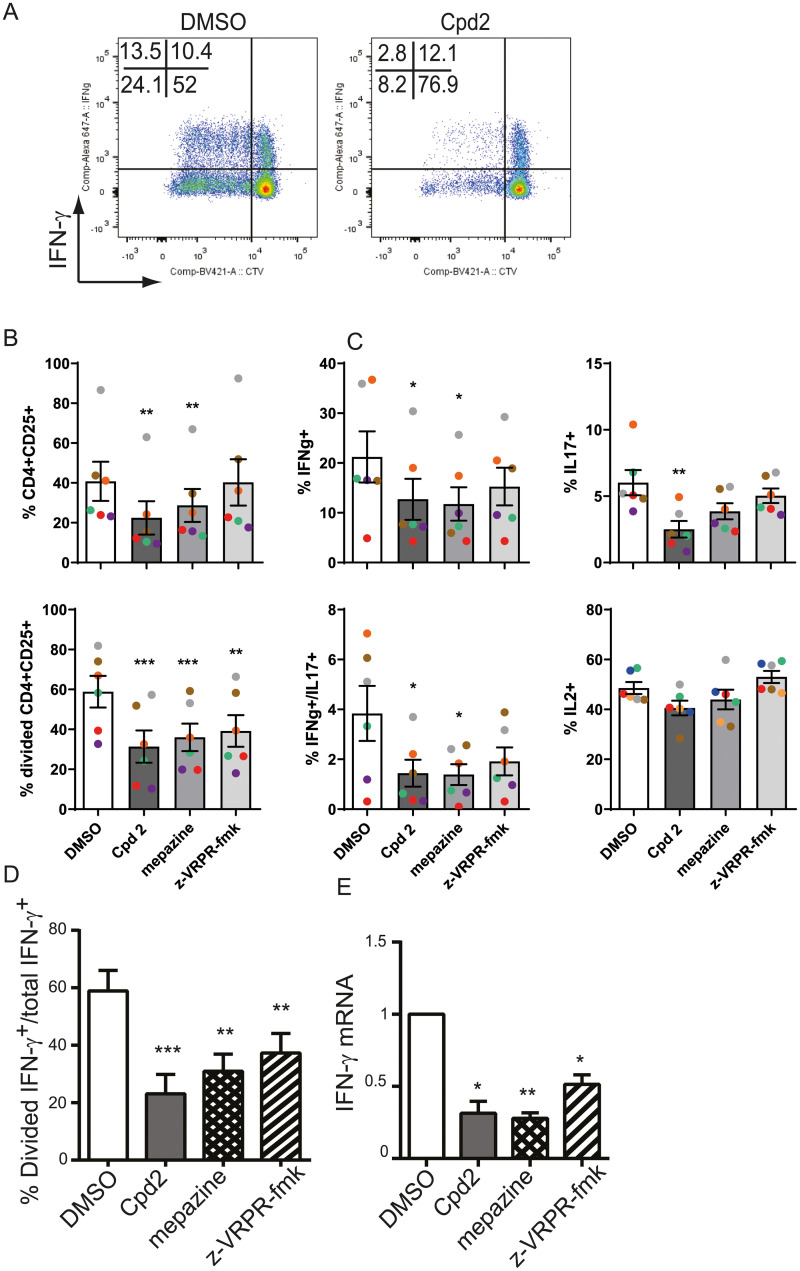
Effect of allosteric MALT1 inhibitors on activation, proliferation and cytokine production of human memory CD4+ CD45RO+ T cells. (A) Representative FACS plots showing IFN-ɣ expression and cell trace violet (CTV) dilution in human memory CD4+ CD45RO+ T cells co-cultured with autologous monocytes and LPS and stimulated with anti-CD3 + anti-CD28 antibodies for 5 days in the absence or presence of 10 μM Compound 2, 5 μM mepazine or 100 μM z-VRPR-fmk. (B) Quantification of the percentage of cells expressing CD25 (top graph) and cells that have divided as defined by CTV dilution (lower graph), (C) and quantification of cells producing either IFN-ɣ, IL-17A, both IFN-ɣ+IL-17A or IL-2. Data are presented as mean ± SEM with n = 6. Coloured dots indicate data from individual donors across different stimulations. (D) Data representing the ratio of CD4+ T cells that have divided and express IFN-ɣ to total IFN-ɣ expressing CD4+ T cells. (E) IFN-ɣ mRNA expression in CD4+ T cells after 3 days culture with 1 μg/mL plate-bound anti-CD3 antibody + 1 μg/mL soluble anti-CD28 antibody was analysed by RT-qPCR. Data is presented relative to DMSO control set at 1 with n = 3 and shown as mean ± SEM. The significance of the data was evaluated by donor-matched one-way ANOVA with Dunnett’s multiple comparison test compared to DMSO control. *:p<0.05; **:p<0.005; ***:p<0.001.

To evaluate the effect of MALT1 inhibitors on effector Th1 and Th17 cells in the inflammatory environment, we stimulated memory CD4+ cells with anti-CD3 antibody plus anti-CD28 antibody in the presence of LPS-treated monocytes. As shown earlier (Fig 3A), MALT1 inhibitors did not interfere with LPS signaling. All MALT1 inhibitors down-regulated proliferation of CD25+CD4+ cells (Fig 5A and 5B). Anti-CD3 antibody plus anti-CD28 antibody stimulation of memory CD4 cells in the inflammatory environment resulted in the activation of IL-2, IFN-γ, IL-17 and both IFN-γ and IL-17 secreting CD4+ memory cells, and allosteric MALT1 inhibitors down-regulated activation and proliferation of IFN-γ, IL-17 and also IFN-γ and IL-17 co-secreting memory CD4+ cells whereas no effect was seen on IL-2 expressing memory CD4+ cells (Fig 5C). We could not see a decrease in the expression level of IFN-γ or IL-17 in the cytokine secreting cells (S3 Fig). This suggests that allosteric MALT1 inhibition modulates IFN-γ and IL-17 response mainly by inhibiting proliferation of the cytokine secreting memory CD4+ cells. However, the percentage of proliferating IFN-γ expressing cells among all IFN-γ expressing cells was significantly decreased in MALT1 allosteric and active site inhibitor treated CD4+ memory cells (Fig 5D) indicating an additional effect of MALT1 treatment on Th1 differentiation. We also showed that the expression of IFN-γ transcripts in memory CD4+ cells activated in the inflammatory milieu was reduced in the presence of MALT1 allosteric and active site inhibitors (Fig 5E), which reflects the reduction in the numbers of IFN-γ expressing cells. Together, these results show that IFN-γ response in human T-cells is down-regulated by MALT1 allosteric inhibitors both in naïve and memory CD4+ cells, and furthermore the activation of effector Th17 and Th1/Th17 cells is dampened. The down-regulation of Th1 response was due to the inhibition of the proliferation of IFN-γ secreting memory CD4+ cells, and as a net effect a decrease in the IFN-γ mRNA expression was observed while the IFN-γ protein production per secreting cell remained unaltered. We conclude that allosteric inhibition did not increase IFN-γ mRNA or protein expression similarly as reported in the mouse model with MALT1 protease inactivation.

### The effect of MALT1 inhibitors on human regulatory T-cells

**Fig 6:**
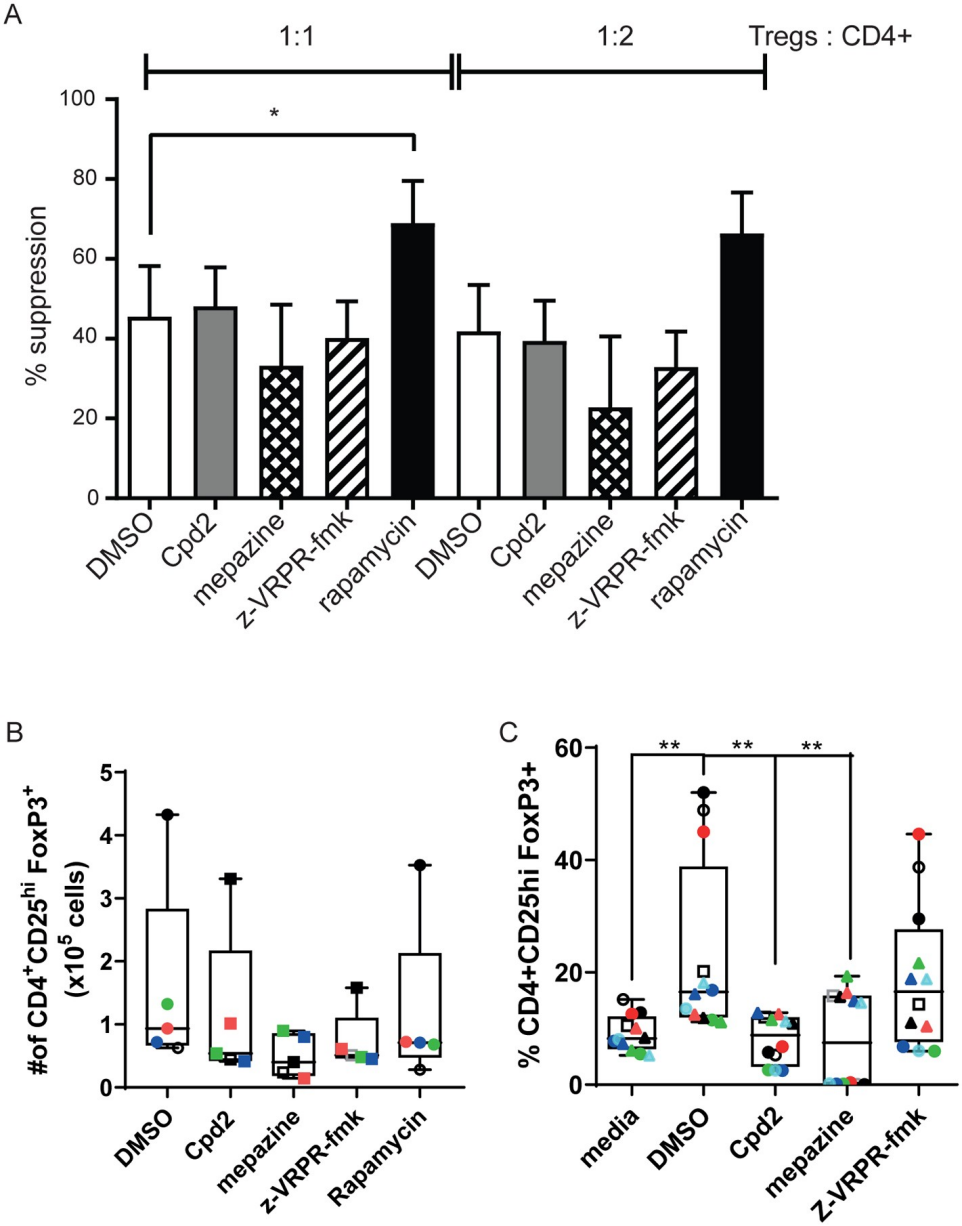
Allosteric MALT1 inhibitors affect *de novo* induction of human Tregs *in vitro* but not the function of *in vitro* expanded blood Tregs. (A) Naïve human Tregs expanded *in vitro* for 14 days in the presence of rapamycin and then treated for 2 days with DMSO (n = 9), Compound 2 (n = 5), mepazine (n = 5), z-VRPR-fmk (n = 5) or rapamycin (n = 9). Expanded and treated Tregs were then co-cultured with CTV labeled CD4+ T cells and stimulated with anti-CD3 at the indicated ratios for 3 days. Suppressive activity was assessed as the reduction in CD4+ responder T cell proliferation normalised to responder cell proliferation in the absence of Tregs. Data is presented as mean ± SEM. (B) Number of CD4^+^CD25^+^FoxP3^+^ human Tregs after 14-days expansion *in vitro* in the presence of Compound 2, mepazine or z-VRPR-fmk or rapamycin (n = 6). Data are presented as Box and whiskers of number of live Tregs. (C) Percentage of *de novo* induced Tregs from naïve human CD4^+^ T cells stimulated for 7 days with anti-CD3 antibody plus anti-CD28 antibody in the absence or the presence of TGFβ + IL-2 + rapamycin in the presence of Compound 2, mepazine or z-VRPR-fmk (n = 5). Media indicates the basal induction of FoxP3 in activated CD4+ T cells in the absence of TGFβ + IL-2 + rapamycin. Coloured dots indicate data from individual donors across different stimulations. Data are presented as Box and whiskers plot. For all experiments, Compound 2 was used at 10 μM, mepazine at 5 μM, z-VRPR-fmk at 100 μM and rapamycin at 100 nM. One-way ANOVA with Dunnett’s for multiple comparison test was used for statistical analyses.**:p<0.01.

As MALT1 protease deficient mice showed dysfunction of their regulatory T-cells, we studied the suppression function and FOXP3 expression of blood-derived human regulatory T-cells (FOXP3+CD4+CD25+) and found that the suppression activity of *ex vivo* expanded regulatory T-cells was not affected by MALT1 inhibition (Fig 6A). Also, absolute numbers,viability and survival of expanded FOXP3+CD4+CD25+ cells were not affected by MALT1 inhibitors (Fig 6B, S5A Fig).

Next, we wanted to evaluate the effect of MALT1 inhibition on the induction of peripheral regulatory T-cells. We differentiated regulatory T-cells from naïve human T-cells *in vitro* using rapamycin, TGF-β and IL-2. Both protease and allosteric inhibitors impaired *in vitro* induction of regulatory T-cells from naïve T-cells (Fig 6C). Together, these data suggest that MALT1 allosteric inhibition does not interfere with the function of circulating human regulatory T-cells but with the induction of Tregs.

### Allosteric MALT1 inhibition decreases B-cell proliferation and antibody production

**Fig 7:**
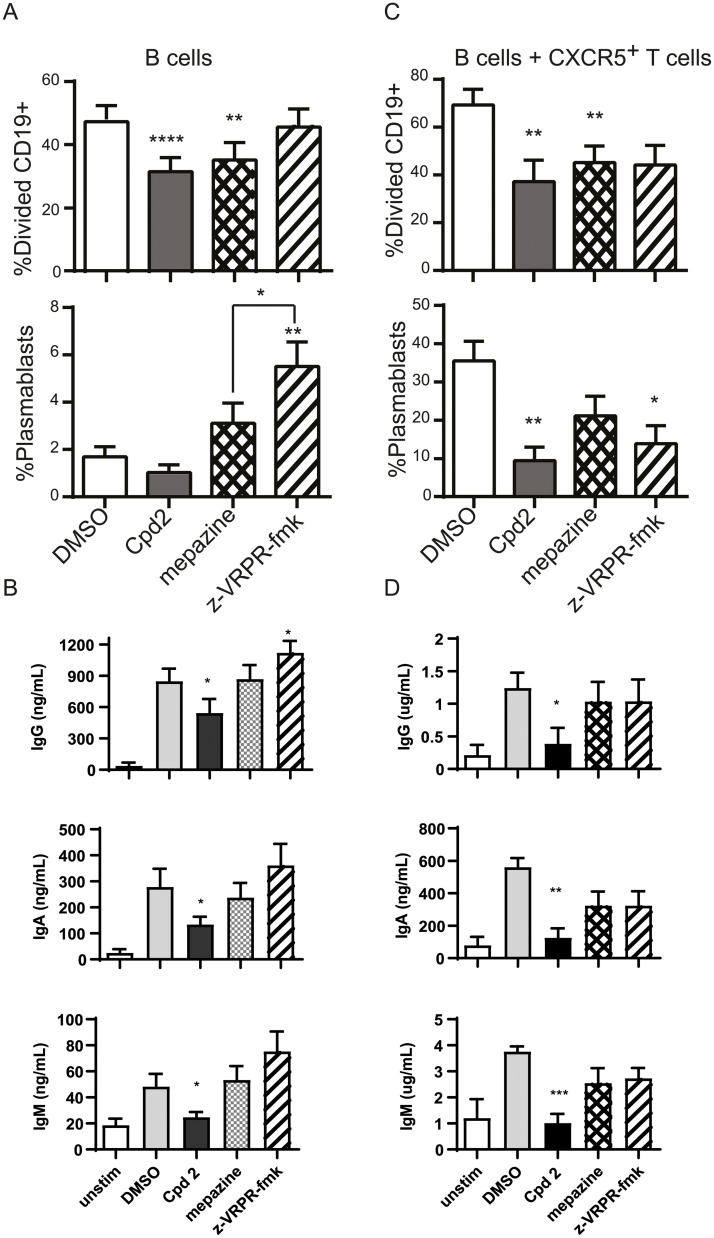
Allosteric MALT1 inhibitor Compound 2 reduces BCR-induced and T-cell induced B-cell immune responses. (A) and (B) CFSE stained CD19^+^ cells were stimulated for 5 days with anti-IgM+anti-CD40 + IL-21 in the presence or absence of 10 μM Compound 2, 5 μM mepazine or 100 μM z-VRPR-fmk. (n = 8). Proliferation (A) was assessed as CFSE dilution of CD19+ B cells, presence of CD19^+^CD27^hi^ CD38^hi^ plasmablasts was measured using flow cytometry and (B) levels of IgG, IgA and IgM in the supernatant culture of unstimulated or stimulated B cells was measured using human MSD multiplex isotyping panel. (C) and (D) CFSE stained human CD19+ cells were co-cultured with FACS sorted CD4^+^CXCR5^+^CD45RO^+^ T-cells and stimulated for 5 days with SEB 100 ng/ml (n = 6) in the presence or absence of 10 μM Compound 2, 5 μM mepazine or 100 μM z-VRPR-fmk. Proliferation (A) was assessed as CFSE dilution of CD19+ B cells, presence of CD19^+^CD27^hi^ CD38^hi^ plasmablasts was measured using flow cytometry and (B) levels of IgG, IgA and IgM in the supernatant culture of unstimulated or stimulated B cells was measured using human MSD multiplex isotyping panel. Data are presented as mean ± SEM. The significance of the data was evaluated by donor-matched one-way ANOVA with Dunnett’s multiple comparison test compared to DMSO control. *:p<0.05; **:p<0.005;****:p<0.0001.

To evaluate the effect of MALT1 inhibitors on B-cell receptor mediated activation, we first studied the effect of MALT1 inhibitors on direct B-cell receptor mediated activation. The proliferation and antibody production of human purified B-cells stimulated by anti-IgM plus anti-CD40 was decreased by allosteric MALT1 inhibition (Fig 7A and 7B), but not active site inhibition, whereas no effect on differentiation of B-cells was seen by allosteric inhibition (Fig 7A). Interestingly, the active site inhibitor z-VRPR-fmk increased differentiation of B-cells to plasmablasts (Fig 7A). Next, we wanted to evaluate the effect of MALT1 inhibitors on T-cell dependent B-cell activation. We co-cultured SEB activated CXCR5+ CD4+ T-memory-cells with B-cells in the presence BAFF, and Compound 2 or z-VRPR-fmk. Compound 2 decreased proliferation, plasmablast differentiation and antibody production while z-VRPR-fmk only affected plasmablast differentiation (Fig 7C and 7D).

### Treatment with MALT1 allosteric inhibitor alleviates cytokine storm and does not enhance IFN-γ production *in vivo*

**Fig 8:**
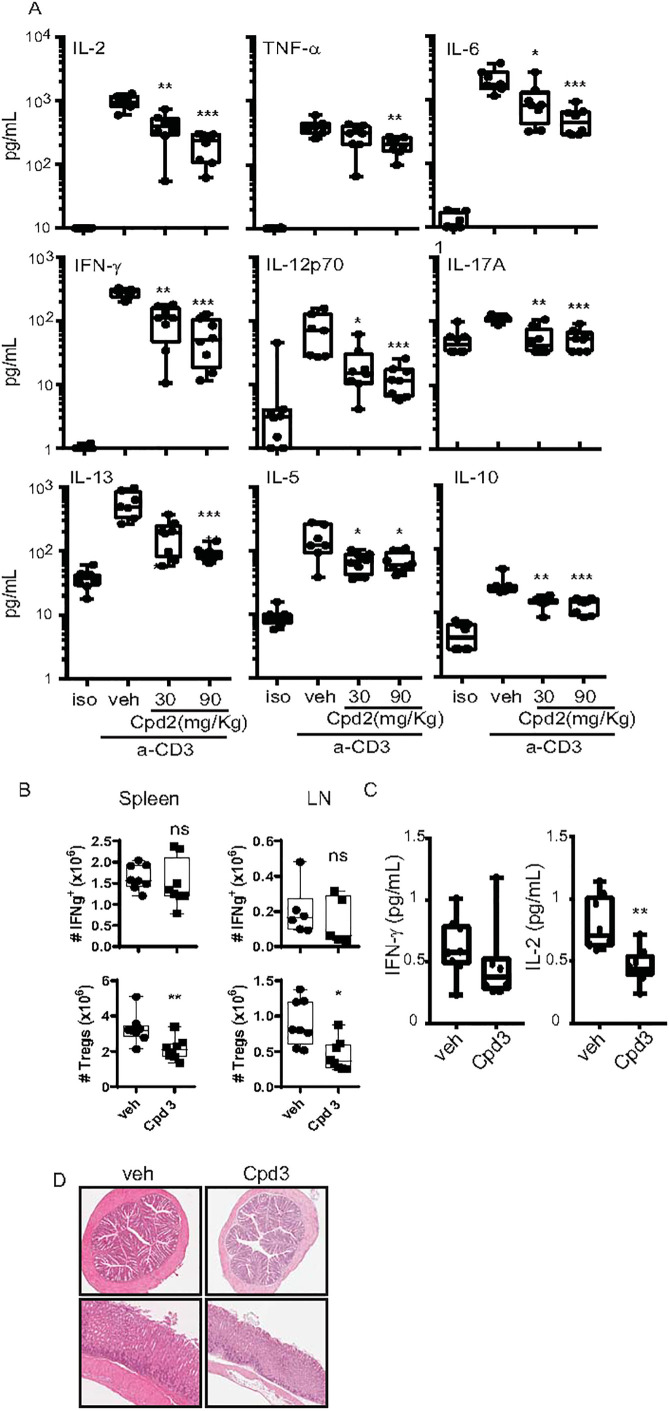
Effect of the allosteric MALT1 inhibitors on anti-CD3 antibody induced cytokines and on IFN-γ producing-CD4+ T cells and FoxP3+Tregs *in vivo*. Blood of C57BL/6 females was collected 4h after anti-CD3 or IgG isotype control antibody injection (i.p) following 30 min pre-treatment with Compound 2 (i.p) at 30 and 90 mg/kg. (A) plasma cytokine levels in C57BL/6 mice 4 h after intraperitoneal anti-CD3 antibody injection following 30 min pre-treatment with 2 at 30 and 90 mg/kg (n = 7–8). (B) C57BL/6 mice treated orally with Compound 3 at 10 mg/kg twice daily for 4 weeks (n = 8 per group). Quantification of numbers of IFN-ɣ producing CD4+ T cells and FoxP3+ CD4+CD25^high^ regulatory T-cells in spleen and lymph nodes (LN) as assessed by flow cytometry. (C) Quantification of plasma IFN-ɣ and IL-2 levels following 4-week treatment with Cpd 3. Cytokines levels were analysed by multiplex MSD analysis. Dots represent individual mice and data is presented as a Box and whiskers plot. (D) Representative H&E-stained colon (top) and stomach fundus (bottom) from 4-week Cpd 3 treated mice. Data were evaluated by unpaired two-sided t test with Welch’s correction against the vehicle group *:p<0.05; **:p<0.01;***:p<0.001.

As mice expressing protease inactive (PD) MALT1 developed an enhanced IFN-γ response, we measured a panel of circulating cytokines in C57BL/6 mice pre-treated first with an allosteric inhibitor and then stimulated with anti-CD3 antibody intraperitoneally to activate T-cells (S4 Fig). We found a significant decrease in all the measured circulating cytokines including IFN-γ (Fig 8A). To study the chronic effects of pharmacological inhibition of MALT1 *in vivo*, we used a recently reported allosteric MALT1 inhibitor from the patent literature, Compound 3 [34] (example 10 in reference 35) (Fig 1, Table 1) with pharmacological properties more suitable for longer-term, oral treatment than compound 2. We treated naïve C57BL/6 mice with 3 (10 mg/Kg, per oral, BID) for four weeks. While percentage and numbers of IFN-γ producing T-cells and circulating IFN-γ levels were not significantly changed, percentage and numbers of FOXP3 positive regulatory T-cells were decreased in spleen and in lymph nodes after treatment (Fig 8B and 8C). Thus, an allosteric MALT1 inhibitor interferes with regulatory T-cell differentiation or survival, as we also observed decreased levels of circulating IL-2 (Fig 8C). However, this effect did not manifest as an activation of the Th1 pathway and circulating IFN-γ levels and no signs of tissue inflammation or lymphocytic infiltrates were observed in all examined tissues in the mice after 4 weeks treatment with Compound 3 (Fig 8D). However longer studies need to be undertaken to exclude the possibility that target-related toxicity that may develop with longer term dosing.

## Discussion

We demonstrate that an allosteric MALT1 inhibitor, Compound 2, which affects both scaffold and protease activity of MALT1, down-regulated *in vitro* human Th1, Th17 and Th1/Th17 effector cell response as well as T-cell dependent B-cell activation without interfering with the suppression function of circulating regulatory T-cells. Furthermore, allosteric MALT1 inhibition did not induce enhanced IFN-ɣ response in human CD4 T-cells *in vitro* or *in vivo* in a mouse model including a short-term model of T-cell activation and a long-term treatment of naïve mice with a MALT1 inhibitor. Thus, the immunomodulatory effect of allosteric MALT1 inhibitors is different from that seen in mice with protease-inactive MALT1 which results in an enhanced IFN-ɣ activation and development of spontaneous autoinflammation [19–22]. This phenotype difference is likely explained by the effect of allosteric MALT1 inhibitor on both scaffold and protease activity of MALT1 as we demonstrate. Our findings support the view implicated from the animal models, in which autoimmune phenotype is associated with the abolishment of MALT1 protease activity, whereas the development of autoimmunity is not seen in MALT1 knock-out (KO) mice [17]. Actually, MALT1 KO and PD mice have been shown to be protected from experimental allergic encephalitis but only PD mice exhibited autoimmune disease due to the role of MALT1 enzymatic activity during Treg development [17]. Our studies indicate that a MALT1 allosteric inhibitor affecting both scaffold and protease activity can be considered as a potential treatment for autoantigen-driven autoimmune pathology due to its effect on TCR- and BCRmediated activation, such as type 1 diabetes and systemic lupus erythematosus.

Furthermore, our data here show that allosteric inhibitors of MALT1 not only decrease activation and proliferation of Th1, but also Th17 and IFN-γ and IL-17 co-secreting cells. Our data indicates that interference with memory Th1/Th17 responses by allosteric MALT1 inhibitors seems to be due to both inhibition of cell proliferation and a direct effect on cell differentiation, as Compound 2 decreased both the percentage of proliferated memory T cells and the percentage of IFN-γ producing cells among proliferated cells. We also observed decreased levels of IFN-γ cytokine mRNA in Compound 2-treated memory T cells, but we cannot conclude whether this is a consequence of decreased RNA synthesis or decreased stability due to decreased MALT1-dependent cleavage of the RNAse Roquin. The effect of MALT1 on Th17 cell differentiation has been clearly demonstrated, and the IKK signaling and canonical NF-κB activation is more important for Th17 cells than for Th1 cells. The absence of MALT1 signaling in mice affects the IL-17 secretion independently of the inhibition of cell proliferation [35]. It seems that MALT1 regulates Th17 cells at functional level too. In MALT1 deficient mice, Th17 cells appeared to be non-pathogenic in the induction of experimental autoimmune encephalitis [36]. This may be related to the fact that that Th1/Th17 plasticity was prevented in MALT1 deficient mice. The mechanisms of MALT1 in the regulation of Th17 immunity are not fully understood, but the data in mouse models suggest that both IKK-induced canonical NF-κB activation and the paracaspase activity of MALT1 are important for Th17 cell differentiation. The cleavage of RELB was impaired in MALT deficient Th17 cells resulting in the altered cellular localization of this protein [36]. Here, we show that both Th17 and Th1/Th17 cell activation is inhibited by an allosteric inhibitor affecting both scaffold and protease function of MALT1, whereas a MALT1 active site inhibitor did not decrease activation of human Th17 effector cells *in vitro*. Our results suggest that pharmacological allosteric inhibition of MALT1 is an attractive approach in the treatment of autoimmune conditions driven by Th17 immunity/plasticity.

MALT1 inhibition down-regulated remarkably dectin-1 induced cytokine secretion by mDCs and monocytes, such as TNF-α, IL-6, IL-1β, IL-23 and IL-10. The C-type lectin dectin-1 has emerged as an important extracellular sensor in fungal recognition by DCs and in the induction of protective Th1 and Th17 immune responses. Dectin-1 deficiency in humans causes susceptibility to mucocutaneous fungal infection. Antifungal immunity through the induction of Th17 responses requires the production of mature, active IL-1β. The role of MALT1 scaffold function has been shown to be important for IL-1β secretion induced by dectin-1, and whereas the CARD9-BCL10-MALT1 scaffold direct IL-β transcription, the recruitment of MALT1-caspase-8 and ASC into this scaffold results in the processing of pro-IL-1β by caspase-8 [36]. MALT1 inhibition thus affects this extracellular dectin-mediated sensing for pathogens through a non-canonical caspase-8-dependent inflammasome activation. The obvious risk related to MALT1 inhibition is thus susceptibility to infections, particularly to fungal infections due to the link between dectin, MALT1, IL-1β and IL-17 [37]. This risk may be further potentiated by the effects of MALT1 inhibition on Th17 cells. We believe that the increased risk for fungal infections has to be subject to clinical monitoring and has to be weighed against possible MALT1 inhibition treatment benefits in patients suffering from autoimmune disease.

Our studies on the effect of MALT1 inhibition on human B-cell responses indicate that MALT1 inhibitors only modestly affect B-cell activation, but down-regulated T-cell dependent B-cell responses, such as B-cell proliferation, antibody production and plasmablast differentiation. This may indicate that the regulation of NF-κB activation by MALT1 is less important for B-cell receptor mediated signalling than T-cell receptor signalling. In chimeric mouse model, the role of MALT1 has been demonstrated to be crucial for B-cell proliferation and differentiation [37]. B-cells may also participate in the presentation of autoantigens and inhibition of the proliferation of B-cells may be beneficial in this mechanism.

We did not find a detrimental effect of MALT1 allosteric inhibition on the expansion, viability, FOXP3 expression or suppression function of human circulating regulatory T-cells *in vitro*. However, MALT1 inhibition impaired the differentiation of regulatory T-cells from naïve CD4 cells, even though exogenous IL-2 was added in the culture. The function of thymus-derived natural regulatory T-cells may thus remain intact by MALT1 allosteric inhibition, whereas the induction of regulatory T-cells in the periphery may be altered. However, recent studies have shed new light on the function of the CARMA1/BCL10/ MALT1 (CBM-complex) in regulatory T cells. Genetic ablation of either BCL10, CARMA1 or MALT1 in Foxp3-expressing cells independently led to autoimmune–like wasting disease in mice [38–40]. In all 3 studies, the authors link the phenotype to decreased expression of effector molecules such as KLRG1, CTLA4 and PD-1 and decreased suppressive capacity of regulatory T cells suggesting that the CBM complex directly regulates Treg function. Interestingly, while mice with MALT1-deficient Tregs and mice with enzymatically-dead MALT1 in Tregs both developed autoimmune-like wasting disease, only full MALT1-KO in Tregs reduced splenic Treg frequencies and increased IFNg/IL17 production in conventional CD4 T cells [40]. While Tregs expressing enzymatically-deficient MALT1 did not exhibit diminished suppressive activity *in vitro* as the MALT1-KO Tregs did [40], treatment of mice with mepazine did inhibit Treg function *in vivo*. Together these data indicate that both MALT1 enzymatic activity and scaffolding function are important for different aspects of Treg function [38,39];. The translation of the data into the clinical situation is challenging as in these experimental systems the CBM complex was deleted during early development and proximal to Treg induction, as soon as cells started expressing Foxp3. In a clinical setting pharmacological inhibition will be achieved in a fully developed immune system and in already differentiated regulatory T cells. Effects of MALT1 inhibition on Treg function might be less severe in this setting and rather accumulate over time. It will thus be important to monitor if and when negative effects of MALT1 inhibition on Treg function will outweigh possible beneficial effects on limiting autoimmune effector T cell proliferation. In a separate study, we have now treated autoimmune NOD mice for up to 6 weeks and have neither observed a significant increase in systemic levels of inflammatory cytokines or deterioration of the underlying autoimmune condition of NOD mice (manuscript in preparation).

Our interpretations of the effects of MALT1 allosteric inhibitor on human immune cells are limited by their *in vitro* nature. However, we used a large panel of different assays for various human immune cells, such as different stimuli for T- and B-cell activation including T-cell dependent B-cell activation, and we studied T-cell responses in the presence of LPS stimulated monocytes in order to mimic the inflammatory milieu in autoimmune conditions. We also performed *in vivo* studies, such as a mouse model of anti-CD3 antibody treatment-induced T-cell activation and a long-term treatment with a MALT1 allosteric inhibitor and we could not see any indicator for increased IFN-γ production or signs of tissue inflammation in the treated mice.

The genetic findings of MALT1 gene mutations also suggest our interpretation that the outcome of the phenotype associated with the disruption of MALT1 depends on the nature of the mutation [41,42]. The loss of function mutations in the MALT1 gene identified in patients with immunodeficiencies have been associated with high risk of infections, but not with autoimmune conditions. In a patient with MALT1 deficiency, 10–15% recovery of MALT1 expressing T-cells was enough to result in clinical improvement [40] However, in a recent study IPEX-type syndrome was reported in individuals with MALT1 mutation [41]. With small molecule inhibitors, the inhibition of MALT1 pathway will be partial, and can be titrated based on dose and dose frequency, and thus we believe that the infection risk related to pharmacological MALT1 inhibitors may not be a significant problem.

In conclusion, our results show that allosteric inhibition of the MALT1 pathway does not lead to increased IFN-γ response despite observing a decrease in Treg numbers following long-term treatment and we do not observe tissue inflammation as seen in MALT1 protease-dead mice. Thus, we consider pharmacological inhibition of MALT1 as a viable target for autoimmune disease mediated by antigens driven activation of Th1/Th17 effector cells and B-cells.

## Supporting information

S1 Fig Gating strategy for identification of human mDCs and monocyte populations. (A) Cell surface markers and gates for identification of mDCs, CD14+ monocytes and CD16+ monocytes in human PBMCs. (B) Representative example of IL-6 and TNF-α expression in a mDC population of PBMCs upon zymosan or LPS stimulation. (C) Representative histograms showing the expression levels of co-stimulatory molecules before and after LPS or zymosan stimulation in a mDC population of PBMCs.
(EPS)

S2 Fig MALT1 inhibition in mDCs and monocytes leads to decreased cytokine production in zymosan-treated cells but not in LPS-treated cells. (A) Expression of different cytokines in the supernatant of *ex vivo* purified 5-hour LPS-treated mDCs in the presence of 10 μM mepazine, 10 μM Compound 2 or 100 μM z-VRPR-fmk. (B, C) Quantification of cytokine levels in the supernatant of *ex vivo* purified 5-hour zymosan- or LPS-treated monocytes (CD14+ or CD16+) in the presence of 10 μM mepazine, 10 μM Compound 2 or 100 μM z-VRPR-fmk. Bars represent means + SD with n = 5 and dots represent individual donors. Values are normalized to cells without MALT1 inhibitors (set to 100%).
(EPS)

S3 Fig Effect of allosteric MALT1 inhibitor Compound 2 on activation, proliferation and cytokine production of human memory CD4+ CD45RO+ T cells. Cell trace violet stained human memory CD4+CD45RO+ cells were co-cultured with autologous LPS-activated monocytes and stimulated for 5 days with 1 μg/mL soluble anti-CD3 antibody + 1 μg/mL soluble anti-CD28 antibody in the presence of 10 μM Compound 2, 5 μM mepazine or 100 μM z-VRPR-fmk. (A) Representative FACS plot of IFN-ɣ and IL-17A expression in human memory CD4+ T cells left untreated or treated with Compound 2 during stimulation. (B) Quantification of IFN-ɣ and IL-17A expression levels as measured using a flow cytometer and expressed as Geometric Mean Fluorescence in human memory CD4+ T cells. Data are presented as mean ± SEM with n = 6. Data was evaluated by donor-matched one-way ANOVA with Dunnett’s multiple comparison test compared to DMSO control.
(EPS)

S4 Fig Pharmacodynamic properties of MALT1 inhibitor Compound 2. Blood of C57BL/6 female mice was collected at the indicated time points 4 h after anti-CD3 antibody injection (i.p) following 30 min pre-treatment with Compound 2 (i.p) at 30 and 90 mg/kg. (A) Quantification of free drug concentration in plasma over time after i.p administration of Compound 2 at 30 and 90 mg/kg respectively. Data is shown as mean ± SD (n = 2). Dotted line indicates EC_50_ Enz and refers to potency of Compound 2 to inhibit enzymatic MALT1 activity in a biochemical assay. (B) Quantification of free drug concentration in plasma plotted against plasma levels of IFN-**γ**4 h after anti-CD3 antibody injection. Symbols represent individual mice and dotted line indicates EC_50_ Enz and refers to potency of Compound 2 to inhibit enzymatic MALT1 activity in a biochemical assay.
(EPS)

S5 Fig Viability of compound-treated Tregs. Naïve human Tregs expanded *in vitro* for 14 days in the presence of rapamycin and then treated for 2 days with DMSO (n = 9), Compound 2 (n = 5), mepazine (n = 5), z-VRPR-fmk (n = 5) or rapamycin (n = 9). (A) Viability of human *in vitro* expanded Tregs as measured by flow cytometry. Data are from 5 donors each and is presented as Box-Whisker plot with median±25th and 75^th^ percentile and range.
(EPS)

S6 Fig Viability of compound-treated Tregs. C57BL/6 mice treated orally with Compound 3 at 10 mg/kg twice daily for 4 weeks (n = 8 per group). (A) Quantification of the percentage of IFN-ɣ producing CD4+ T cells and FoxP3+ CD4+CD25^high^ regulatory T-cells in spleen and lymph nodes (LN) as assessed by flow cytometry. Dots represent individual mice and data is presented as a Box and whiskers plot. Unpaired two-sided t-test with Welch’s correction against the vehicle group was used to determine statistical significance *:p<0.05; **:p<0.01; ***:p<0.001.
(EPS)

S1 File Fig 2 raw data. (XLSX)

S2 File Fig 3 raw data. (XLSX)

S3 File Fig 4 raw data. (XLSX)

S4 File Fig 5 raw data. (XLSX)

S5 File Fig 6 raw data. (XLSX)

S6 File Fig 7 raw data. (XLSX)

S7 File Fig 8 raw data. (XLSX)

S8 File S2 Fig raw data. (XLSX)

S9 File S3 Fig raw data. (XLSX)

S10 File S4 Fig raw data. (XLSX)

S11 File S5 Fig raw data. (XLSX)

S12 File S6 Fig raw data. (XLSX)

S1 Raw images (PDF)
