# Supplementary figures and images for: A MALT1 inhibitor suppresses human myeloid DC, effector T-cell and B-cell responses and retains Th1/regulatory T-cell homeostasis
Source: PLoS One. 2020 Sep 1;15(9):e0222548. doi: 10.1371/journal.pone.0222548 (PMC7462277; doi:10.1371/journal.pone.0222548)

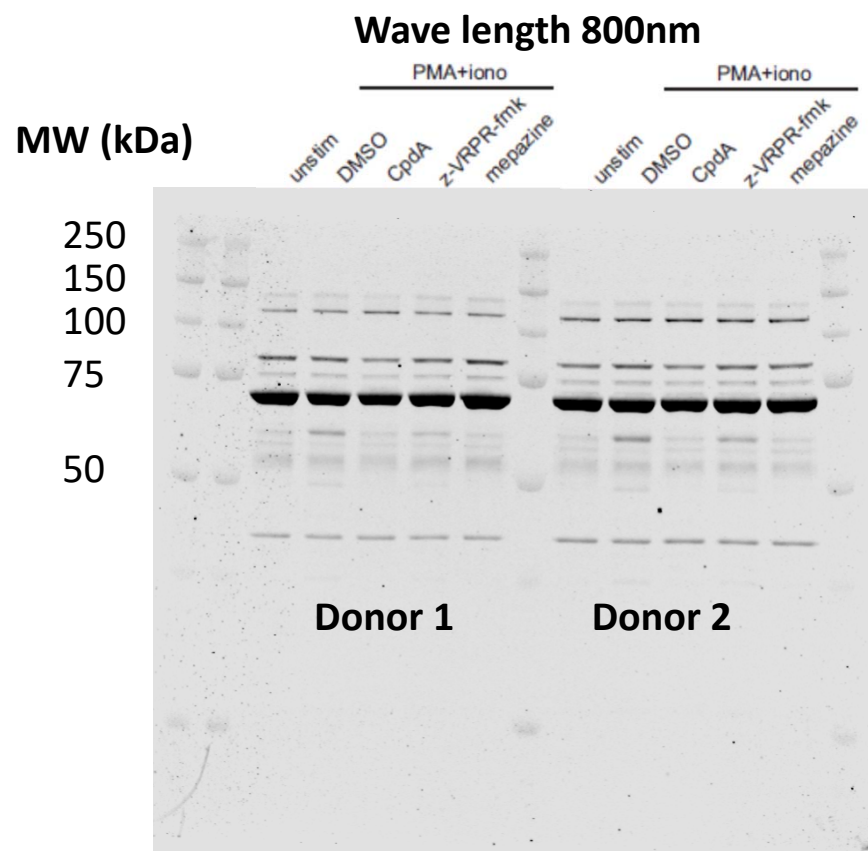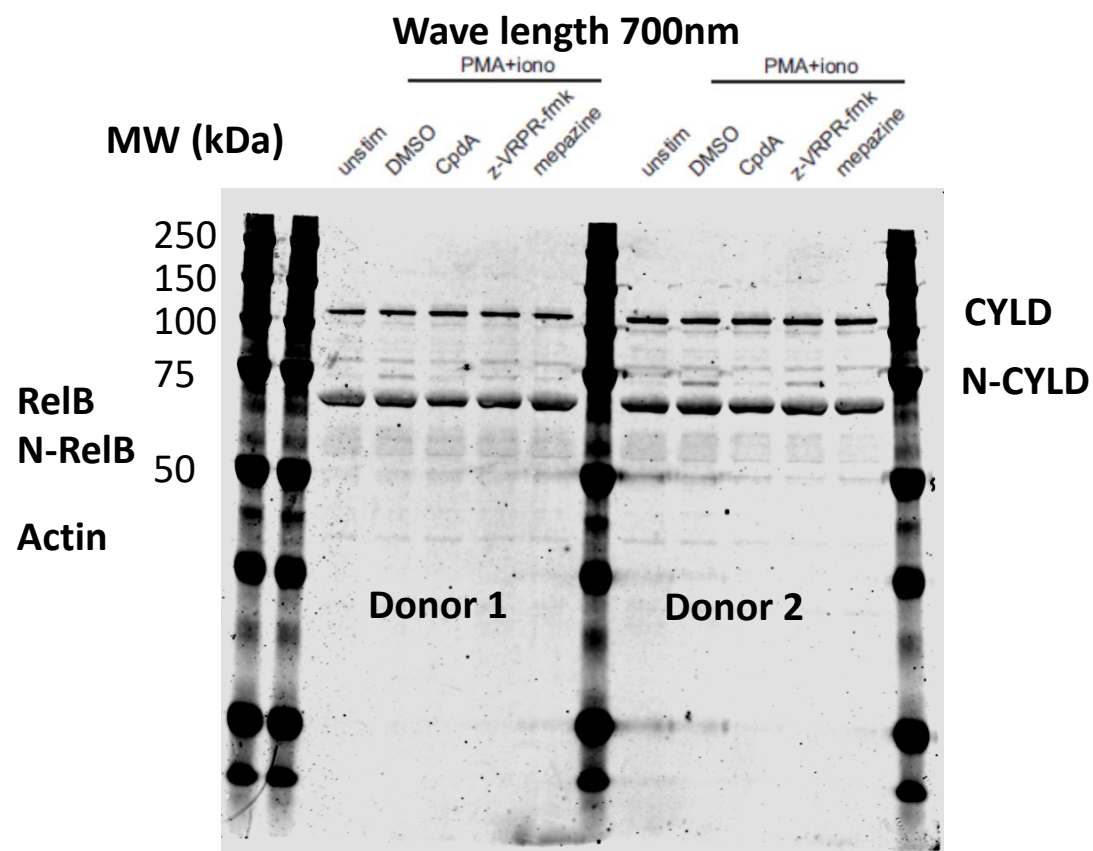

Supplement: S1 Raw images — (PDF) [file pone.0222548.s019.pdf]
